# Protectin DX as a therapeutic strategy against frailty in mice

**DOI:** 10.1007/s11357-023-00789-3

**Published:** 2023-04-14

**Authors:** Laís R. Perazza, Adam C. Gower, Holly M. Brown-Borg, Paola Divieti Pajevic, LaDora V. Thompson

**Affiliations:** 1 Department of Physical Therapy, Boston University, Boston, MA USA; 2 Clinical and Translational Science Institute, Boston University, Boston, MA USA; 3 Department of Basic Sciences, University of North Dakota School of Medicine and Health Sciences, Grand Forks, ND USA; 4 Department of Translational Dental Medicine, Goldman School of Dental Medicine, Boston University, Boston, MA USA

**Keywords:** Aging, Frailty, Physiological decline, Protectin DX, Liver transcriptomics, Bone mineral density

## Abstract

Frailty in aging is driven by the dysregulation of multiple biological pathways. Protectin DX (PDX) is a docosahexaenoic acid (DHA)-derived molecule that alleviates many chronic inflammatory disorders, but its potential effects on frailty remain unknown. Our goal is to identify age-related impairments in metabolic systems and to evaluate the therapeutic potential of PDX on frailty, physical performance, and health parameters. A set of 22-month-old C57BL/6 male and female mice were assigned to vehicle (Old) or PDX daily gavage treatment for 9 weeks, whereas 6-month-old (Adult) mice received only vehicle. Forelimb and hindlimb strength, endurance, voluntary wheel activity and walking speed determined physical performance and were combined with a frailty index score and body weight loss to determine frailty status. Our data shows that old vehicle-treated mice from both sexes had body weight loss paralleling visceromegaly, and Old females also had impaired insulin clearance as compared to the Adult group. Aging was associated with physical performance decline together with higher odds of frailty development. There was also age-driven mesangial expansion and glomerular hypertrophy as well as bone mineral density loss. All of the *in vivo* and *in vitro* impairments observed with aging co-occurred with upregulation of inflammatory pathways and Myc signaling as well as downregulation of genes related to adipogenesis and oxidative phosphorylation in liver. PDX attenuated the age-driven physical performance (strength, exhaustion, walking speed) decline, promoted robustness, prevented bone losses and partially reversed changes in hepatic expression of Myc targets and metabolic genes. In conclusion, our data provides evidence of the beneficial therapeutic effect of PDX against features of frailty in mice. Further studies are warranted to investigate the mechanisms of action and the potential for human translation.

**Supplementary information:**

The online version contains supplementary material available at 10.1007/s11357-023-00789-3.

## Introduction

As the population ages in the 21^st^ century, frailty is one of the most serious challenges [1, 2]. Frailty is featured by declines in physiological reserves and failure to maintain homeostasis, resulting in increased vulnerability to stressors [3]. It is known that lifestyle factors, such as poor dietary habits, disabilities, loneliness and sedentarism are determinants for frailty and systemic dysfunction [4–6]. Although frailty is often reported in the older adult, the initiation of frailty is quite variable across the lifespan [2]. The heterogeneity and complexity of the physiological decline that causes frailty likely contribute to these variabilities. Thus, in order to elucidate the risk of an older adult to develop frailty, it is important to fully understand all physiological aspects of frailty and how they are connected to each other.

Fried and colleagues identified a phenotype (a.k.a. the physical frailty phenotype) that is a useful clinical assessment tool to standardize criteria for frailty diagnosis [3]. The authors identify frail adults as those in whom three or more of the following criteria are present: unintentional weight loss, self-reported exhaustion, weakness (measured by grip strength), slow walking speed, and low physical activity [3]. The frailty index, another tool that is used extensively to identify frail individuals, was characterized by Mitnitski and colleagues, who postulated that aging correlates with multiple health deficits that culminate in frailty when summed [7, 8]. These two frailty assessment tools identify the physical performance and physical appearance characteristics of an individual, which may in turn reflect consequences of decline in many physiological systems. The physical frailty phenotype and frailty index were reverse-translated to animal models, facilitating meaningful interactions between basic, preclinical and clinical research [9–11].

In an effort to investigate and elucidate the multitude of pathways that drive or underlie physiological decline with aging, the “Hallmarks of Aging”, a conceptual framework, was purported. There is emerging application of the nine hallmarks, investigating either one pathway or biological process involved (e.g., epigenetic alterations, cellular senescence), to tease out the underlying factors contributing to aging and frailty [12]. As frailty is a systemic dysfunction in which inter-organ communication plays an important role, the hallmark focused on inflammation stands out as the most common pathological condition affecting multiple systems at once, and is therefore likely a key process in the initiation of frailty.

The long-chain omega-3 polyunsaturated fatty acids (ω-3 PUFAs) eicosapentaenoic acid (EPA) and docosahexaenoic acid (DHA) are known to promote the active resolution of inflammation, restoring tissue homeostasis [13, 14]. Failure to resolve inflammation is involved in a variety of diseases, such as diabetes, cardiovascular disease (CVD), and chronic kidney disease [15, 16]. Importantly, lipid mediators derived from these ω-3 PUFAs or arachidonic acid (AA), the so-called specialized pro-resolving mediators (SPMs), are described as potent endogenous counter-regulators of inflammation [17]. Protectin DX (PDX), an SPM derived from DHA [18], is potent against many acute and chronic inflammatory disorders, such as end-stage renal disease [19], insulin resistance [20] and arthritis [21]. PDX exerts a beneficial impact on structure, alleviating glomerular fibrosis in end-stage renal disease [19] and maintaining the integrity of lung epithelium in an LPS-induced model of acute respiratory distress syndrome [22]. PDX also preserves tissue functionality, counter-regulating inflammation by enhancing phagocytosis of macrophages in sepsis [23], inhibiting the NLRP3 inflammasome pathway in collagen-induced arthritis [21], increasing alveolar fluid clearance in rats in acute respiratory distress [24], and inhibiting transforming growth factor-β1 (TGF-β1), favoring epithelial wound healing [25]. Lastly, PDX ameliorated insulin sensitivity [20] and inhibited platelet aggregation [26], maintaining physiological functions and delaying disease progression, as evidenced by the efficacy of PDX in reducing mortality in sepsis [23] and in end-stage renal disease [19]. Considering that structure, functionality and resilience to stress (disease) are pivotal in the onset of frailty, we postulated that PDX would be an excellent candidate for the therapeutic delay of frailty. Regarding the complexity and the multiplicity of biological systems that regulate aging and frailty development, we also took the opportunity to confirm and add important remarks to age-related impairments using adult and old female and male mice with a difference in ages of 16 months.

In the current study, daily gavage with PDX for 9 weeks partially reversed some age-associated changes in liver gene expression, alleviated some aspects of physical decline, and promoted robustness. PDX further prevented density loss in both cortical and trabecular bone in female mice. Aging in both sexes correlated with lean and fat mass loss and the presence of visceromegaly. In females, advanced age resulted in impaired insulin clearance as well as in the activation of pro-inflammatory and apoptotic gene sets in the liver. In the kidney, aging was associated with glomerular impairments such as hypertrophy and mesangial expansion. The age-driven declines in physical performance and health parameters increased the risk of frailty development. Overall, the data indicates PDX as a potential therapeutic candidate to delay the frailty onset and the data contributes to the understanding of age-related impairments in a mouse model of aging.

## Material and methods

### Animals

All animal experiments were approved by the Institutional Animal Care and Use Committee (IACUC) at Boston University. C57BL/6 mice were obtained from the National Institute on Aging (NIA) and group-housed in a pathogen-free facility at 20–23°C under a 12-h light to dark cycle with food (Chow diet, Prolab® IsoPro® RMH 3000) and water ad libitum. Animals requiring single housing (e.g., due to fighting) were excluded from the study. Six-month-old female (*n*=15) and male (*n*=15) mice were assigned to the reference mature Adult group. Twenty 22-month-old female and 22-month-old male mice were assigned to the Old group and divided into two groups according to the treatment: vehicle or Protectin DX (PDX). At the end of the experimental timeline, mice were anesthetized with isoflurane and euthanized by cardiac puncture. Gonadal, retroperitoneal, and inguinal white adipose tissues (gWAT, rpWAT, and iWAT, respectively), brown adipose tissue (BAT), liver, kidney, tibialis anterior (TA), extensor digitorum longus (EDL), soleus and gastrocnemius muscles, spleen, pancreas and caecum content were collected and weighed. The tibiae were also harvested. Because of the labor-intensiveness within the experimental design the Adult and Old mice (both treatment groups) were investigated in batches. Animals from each batch were euthanized on the same day from 8 AM to 5 PM, and the order of euthanasia was alternated by group and sex.

### Experimental strategy

To evaluate the therapeutic potential of PDX on frailty and to identify age-related changes in metabolic and musculoskeletal systems, we determined physical performance and other health-related parameters before (baseline) and after therapeutic treatment (endpoint) (Supplemental Figure 1). Forelimb and hindlimb strength, endurance, voluntary wheel activity and walking speed were used to determine physical performance (Supplemental Figure 1). The other health-related *in vivo* parameters obtained were the frailty index (FI), oral glucose tolerance test (oGTT) and electrocardiogram (ECG) (Supplemental Figure 1). The therapeutic treatment consisted of 9 weeks of daily oral gavage with either corn oil (120 μL, Mazola® 100% pure, cholesterol free) as vehicle (Old, *n*=18-19 and Adult *n*=15) or PDX in corn oil (30 ng/g of BW; Cayman Chemical Company, *n*=12-16). Gavaging occurred from 8-11 AM and animals were gavaged in an alternating group and sex order. Weekly body weight (BW), body temperature (BT) and food intake were assessed during the entire experimental timeline.

### Frailty index

The Frailty index (FI) was assessed during the experimental timeline at weeks 4 (baseline) and 13 (endpoint) from 1-5 PM using a deficit rating scale [27], alternating the experimental groups and sexes. Briefly, we used an adapted version of the 31-deficit criteria for FI described by Whitehead and colleagues [28], excluding forelimb grip strength, tail stiffening, BT and BW. Herein, 27 health deficits, including vestibular disturbance, alopecia, dermatitis, coat condition, vision and hearing loss, etc., were evaluated blindly by the same rater. For each deficit, a score of 0 was applied when no sign of deficit was noted, a score of 0.5 for a mild deficit and a score of 1 for a severe deficit.

### Physical frailty phenotype

The physical frailty phenotype criteria were assessed during the experimental timeline at weeks 3 (baseline) and 12 (endpoint) and included the evaluation of 4 abilities: walking speed, strength, endurance and voluntary physical activity as previously described [9]. Specifically, the physical frailty phenotype was assessed over an 8-day period (Supplemental Figure 1) with the initiation of walking speed at day one between 1- 5 PM, followed by strength evaluation on day 2 between 8-11 AM and endurance between 1-5 PM. Voluntary physical activity was assessed by 5 consecutive days of 24hr monitoring initiated between 8-9 AM of day 3 and terminated at 8-9 AM of day 8. Animals were selected in alternating order with respect to experimental group and sex for the experiments mentioned above. The same evaluator was responsible for testing all animals for a given parameter. Except for the voluntary physical activity (activity cages), all physical phenotype evaluations were assessed blindly.

- *Walking speed*

Walking speed was recorded with a rotarod (PanLabLetica Rota-Rod L/S, Catalonia, Spain). Once in the rotarod, mice were maintained at a constant speed of 4 rpm for one minute (acclimation.) After acclimation, the speed was increased 1 rpm every 8 seconds up to 40 rpm over 5 minutes. The walking speed test continued until the mouse was unable to keep up with the speed of the rotarod. A total of three trials were performed for each mouse, with a 10-minute rest period in-between each. The best maximal speed out of the three trials was used as each mouse’s walking speed.

- *Strength*

Strength was recorded using a grip strength meter (Grip Strength Test P/N 760483, Coulbourn Instruments, Whitehall PA) and reported as maximal strength. Briefly, the mouse was gently lowered over the top of the grid so that both its front and hind paws could grip the grid. Once gripped, the tail of the mouse was pulled back steadily so that the mouse’s torso was kept horizontal by holding the base of the tail between thumb and forefinger. Once the mouse was no longer able to maintain its grip, the trial was over, and the maximal grip strength was recorded. Five trials of forelimb and hindlimb strength measures were taken with a 10-minute rest in between each trial. The highest and lowest strength trials were eliminated, and an average was generated with the 3 remaining trials.

- *Endurance*

Endurance was determined using a six-lane motorized treadmill (Exer 3/6 Treadmill; Columbus Instruments, Columbus, OH). The protocol was initiated by a 5-minute warm-up period at a speed of 5 m/min in a non-inclined running deck. Following these 5 minutes, speed was increased 1 m/min every minute. Encouragement to continue with the fatigue test was provided by gently tapping the backside of any mouse that began lagging behind on the treadmill using a cotton stick. Distance to exhaustion in meters was recorded after the third consecutive time the mouse needed extra encouragement.

- *Voluntary physical activity*

Physical activity was recorded using individual activity cages containing a voluntary running wheel (Lafayette Instrument, Indiana, USA) with food and water ad libitum. The test consisted of 1 acclimation day followed by 4 experimental days, in which the total running distance was measured, recorded using scurry activity monitoring software (Lafayette Instrument, Indiana, USA), and reported as kilometers/day. Animals were observed for 20 minutes when returning to the group housing in order to prevent fighting injury.

### Frailty classification

The FI score, the four physical frailty phenotype abilities (walking speed, strength, endurance and voluntary wheel activity) and BW loss were used as criteria to determine the frailty status of each mouse (total of 6 criteria.) For each criterion, baseline data from male and female Old groups were used to calculate the cutoff value by sex. Once determined, these cutoff values were applied to sex-matched Adult and Old groups at both baseline and endpoint. Mice that fell below the 20th percentile for each frailty criterion were considered positive for that marker. Three or more positive frailty markers identified an animal as frail, two positive markers as prefrail, and one or zero as robust.

### Electrocardiogram

At weeks 4 (baseline) and 13 (endpoint) during the experimental timeline, mice were anesthetized with isoflurane and subjected to electrocardiogram (ECG). The surface ECG signal (lead II via limb electrodes) was acquired using a 4 Channel Small Animal ECG/EMG Recorder with LabScribe (IX-BIO4-SA, iWorx Systems, Inc.). Briefly, mice were placed in supine position on a heated platform with the paws connected to the electrode needles. Body temperature was maintained between 35 to 38°C. In order to preserve heart rate between 300 to 370 bpm, isoflurane was vaporized at 3% for induction and 1% for maintenance at a flow rate of 0.4-0.8 liter/min. ECG recordings were initiated after a 3-minute acclimation period and were acquired for a 5-minute duration.

### Analytical methods for oGTT and inflammatory cytokines

During the experimental timeline at weeks 4 (baseline) and 13 (endpoint), an oral glucose tolerance test (oGTT) was performed following a 6-hour fast. Briefly, glucose homeostasis was evaluated after gavage with 1 g/kg of BW of a 50% dextrose solution. Blood was drawn from the saphenous vein (One Touch Ultra glucometer, LifeScan, Inc.) at 0, 15, 30, 60, 90, and 120 minutes following gavage to determine glycemic levels. Insulin and C-peptide concentrations were measured in plasma collected during the oGTT via ELISA assays (Alpco Mouse Ultrasensitive Insulin kit and Crystal Chem Mouse C-Peptide kit, respectively).

Circulating inflammatory cytokines levels were determined in plasma drawn via cardiac puncture during euthanasia. Interferon-γ (IFN-γ), interleukin-10 (IL-10), interleukin-1β (IL-1β), interleukin-6 (IL-6), monocyte chemoattractant protein-1/ (MCP-1/CCL2), regulated on activation, normal T cell expressed and secreted (RANTES/CCL5), and tumor necrosis factor α (TNF-α) were quantified using an immunology multiplex assay (MILLIPLEX® MAP Mouse Cytokine/Chemokine Magnetic Bead Panel, MilliporeSigma). The lowest point of the standard curve was assigned to those samples with a concentration below the detection threshold of the MILLIPLEX assay.

### RNA isolation

Total RNA was isolated using miRNeasy® Mini Kit (Qiagen) according to the manufacturer's protocol. Briefly, 50-60 mg of crushed frozen liver from female mice were homogenized in QIAzol lysis reagent (RNeasy Plus Mini Kit, Qiagen) and then transferred to a RNeasy® Mini column to be further processed with a genomic DNA elimination step by following the manufacturer’s RNA isolation protocol. Total isolated RNA was tested to fit high quality and integrity standards, as determined by the 28S/18S rRNA peak and area ratios (ranges 1.8-2.1 and 1.9-2.5, respectively) and the RNA integrity number (RIN, range 8.2-8.7) using RNA 6000 Pico Assay RNA chips run in Agilent 2100 Bioanalyzer (Agilent Technologies, Palo Alto, CA, USA).

### RNA sequencing analysis

200 ng of total RNA from the liver was used for mRNA-seq library preparation using the NEBNext Poly(A) Magnetic Isolation Module and NEBNext Ultra Directional RNA Library Prep Kit (New England Biolabs, Ipswich, MA, USA). 50 ×50 base paired-end sequencing was performed using a NextSeq 2000 instrument (Illumina, San Diego, CA). FASTQ files were aligned to mouse genome build mm10 using STAR [29] (version 2.6.0c). Ensembl-Gene-level counts for non-mitochondrial genes were generated using featureCounts (Subread package, version 1.6.2) and Ensembl annotation build 100 (uniquely aligned proper pairs, same strand). Separately, SAMtools (version 1.9) was used to count reads aligning in proper pairs at least once to either strand of the mitochondrial chromosome (chrM) or to the sense or antisense strands of Ensembl loci of gene biotype "rRNA" or of non-mitochondrial RepeatMasker loci of class "rRNA" (as defined in the RepeatMasker track retrieved from the UCSC Table Browser). FASTQ quality was assessed using FastQC (version 0.11.7), and alignment quality was assessed using RSeQC (version 3.0.0).

Variance-stabilizing transformation (VST) was accomplished using the varianceStabilizingTransformation function in the DESeq2 R package (version 1.23.10) [30]. Principal Component Analysis (PCA) was performed using the prcomp R function with variance stabilizing transformed (VST) expression values that were z-normalized (set to a mean of zero and a standard deviation of one) across all samples within each gene. Differential expression was assessed using the Wald test implemented in the DESeq2 R package. Correction for multiple hypothesis testing was accomplished using the Benjamini-Hochberg false discovery rate (FDR). Human homologs of mouse genes were identified using HomoloGene (version 68). All analyses were performed using the R environment for statistical computing (version 3.6.0).

Gene Set Enrichment Analysis (GSEA) (version 2.2.1) [31] was used to identify biological terms, pathways and processes that were coordinately up- or down-regulated within each pairwise comparison. The Entrez Gene identifiers of the human homologs of all genes in the Ensembl Gene annotation were ranked by the Wald statistic computed for each pairwise comparison. Ensembl Genes matching multiple mouse Entrez Gene identifiers, and mouse genes with multiple human homologs (or vice versa), were excluded prior to ranking, so that the ranked list represents only those human Entrez Gene IDs that match exactly one mouse Ensembl Gene. Each ranked list was then used to perform pre-ranked GSEA analyses (default parameters with random seed 1234) using the Entrez Gene versions of the Hallmark gene sets obtained from the Molecular Signatures Database (MSigDB), version 7.5.1 [31]. The data discussed in this publication have been deposited in NCBI's Gene Expression Omnibus (GEO) and are accessible through GEO Series accession number GSE228901 (https://www.ncbi.nlm.nih.gov/geo/query/acc.cgi?acc=GSE228901).

### Histological preparations of kidney and skeletal muscles

At euthanasia, the inferior half of the right kidney, EDL, and soleus from male and female mice were isolated and placed in a base mold filled with optimum cutting temperature (OCT) embedding medium and snap-frozen in liquid nitrogen. Samples were stored in -80°C for future analysis. Quantitative mesangial cell expansion was estimated in 10-μm kidney sections stained with periodic acid Schiff stain (PAS) and hematoxylin-eosin (Periodic Acid-Schiff Kit, Sigma, 395B). Briefly, images were acquired in a 40X view and the relative number of pixels of the mesangium was divided by the total area of each glomerulus by using a binary threshold in Image J (V. 1.51j8, NIH, USA). Single fiber cross-sectional area (CSA) in the soleus and EDL muscles was analyzed in 10-μm sections stained with hematoxylin and eosin (Hematoxylin and Eosin Stain Kit, ScyTek laboratories Inc.). Fiber CSA consisted of perimeter measure of an average of 100 fibers from 2-3 slides per sample, using Image J (V. 1.51j8, NIH, USA).

### DEXA (Dual X-ray Absorptiometry)

Bone mineral density (BMD) of the right tibia of both male and female mice was estimated with a dual-energy X-ray absorptiometry (DEXA system, GE Lunar PixiMus I). Briefly, the tibia was harvested at euthanasia and fixed in 4% paraformaldehyde for future analysis. Samples were then transferred into 1X Phosphate-Buffered Saline (PBS) solution to proceed with the scan. Tibia samples were scanned in batches of 10-12 bones for the determination of both trabecular and cortical BMD.

### Micro-CT scanning

Assessment of bone morphology and microarchitecture was performed using micro-computed tomography (μCT40, Scanco Medical, Brüttisellen, Switzerland). In brief, right tibia bones were scanned (10μm^3^ voxel size, 70 kVp peak potential, 113 μA current, and 200 msec integration time) to assess both trabecular and cortical bone microarchitecture. Trabecular bone was contoured in a 1mm long region of interest in the proximal metaphysis, segmented with a threshold of 400 mgHA/cm^3^, and analysis was performed in the Scanco Evaluation program to measure trabecular bone volume fraction (Tb.BV/TV, %), bone mineral density (Tb.BMD, mgHA/cm^3^), trabecular thickness (Tb.Th, μm), trabecular number (Tb.N, mm^-1^), trabecular separation (Tb.Sp, μm) and connectivity density (Conn.D, mm^-3^). Cortical bone architecture was analyzed in the tibial diaphysis in a region beginning 2mm superior to the distal tibiofibular junction and extending 500μm distally, with outcomes including cortical thickness (Ct.Th,μm), cortical tissue mineral density (Ct.TMD, mgHA/cm^3^), bone area fraction (bone area (Ct.Ar)/total area (Tt.Ar, %)) and polar moment of inertia (pMOI, mm^4^).

### Statistical analysis

Data are expressed as mean ± SEM. BW gain, BT change, oGTT, insulinemia during oGTT, plasma C-peptide during oGTT, C-peptide/Insulin ratio during oGTT, physical performance tests, FI (27 health deficits) and electrocardiography data were statistically compared using two-way repeated measures ANOVA with a Bonferroni *post hoc *test (Sigmaplot, USA). Differences on survival rate were determined by Logrank (Mantel-Cox) test (GraphPad, USA). The area under the curve (AUC) was calculated for all curves using GraphPad. All other comparisons were made using T-student tests*.* All results were considered statistically significant at *P* < 0.05.

## Results

### Aging is associated with visceromegaly paralleling fat mass loss

**Fig. 1:**
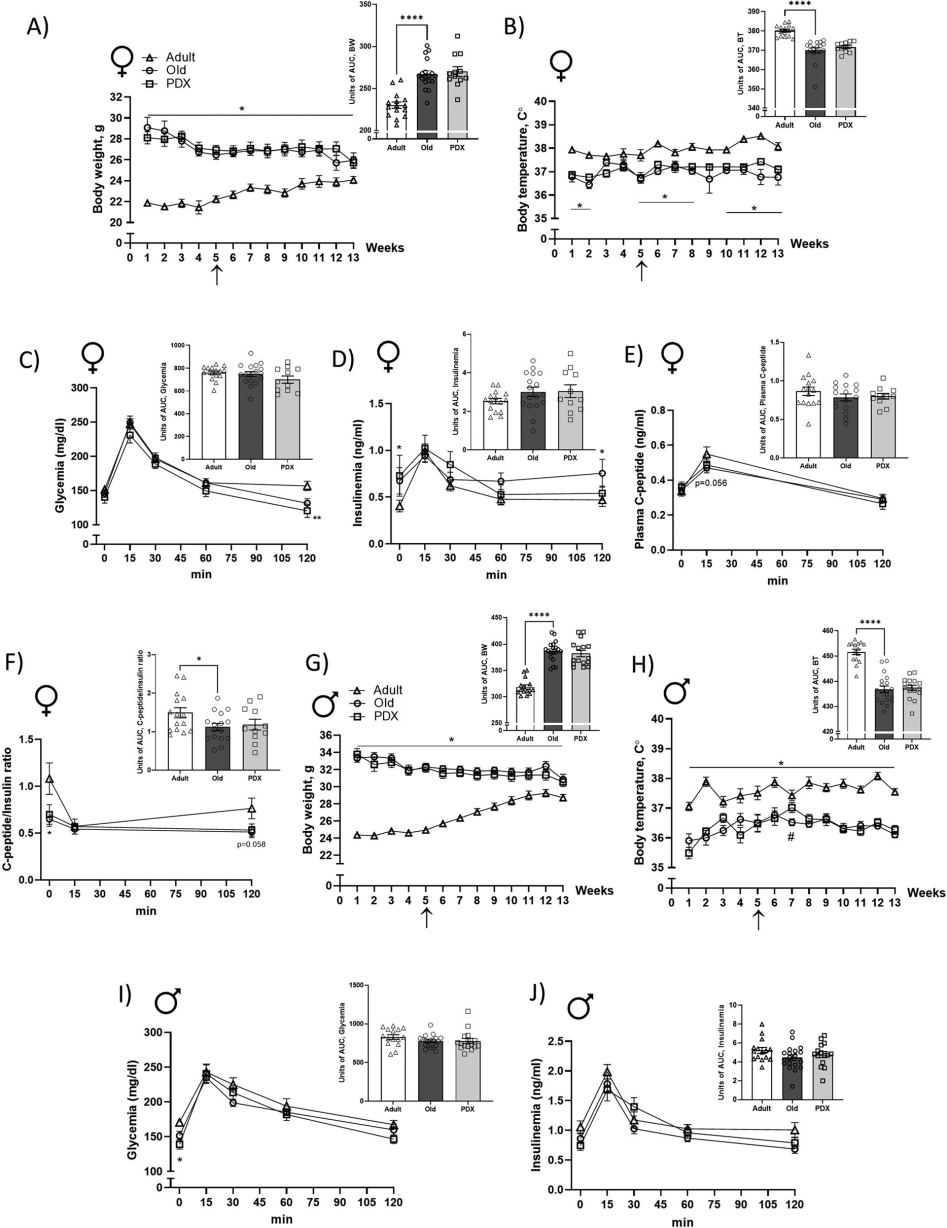
Aging is associated with declines in body weight and temperature in both sexes and reduced insulin clearance in females. Weekly body weight (BW, **A**) and body temperature (BT, **B**) with area under the curve (AUC, insert) of female mice throughout the 13 weeks of the intervention timeline. At week 1 the Adult and Old animals were 6 months and 22 months old, respectively. Plasma levels of glycemia (**C**), insulinemia (**D**) and C-peptide (**E**) during oral glucose tolerance test (oGTT) performed at week 13 with AUC. Plasma C-peptide/insulin ratio (**F**) during oGTT with AUC. Weekly body weight (BW, **G**) and body temperature (BT, **H**) with AUC of male mice throughout the 13 weeks of experimental timeline. Plasma levels of glycemia (**I**), insulinemia (**J**) during oral glucose tolerance test (oGTT) performed at week 13 with AUC. Data are expressed as mean ± SEM. Statistical analysis from BW, BT and oGTT data were determined by repeated measures two-way ANOVA with Bonferroni’s *post hoc* test, whereas AUC differences were calculated by Student’s *t* test. *P < 0.05 vs. Adult; ****P < 0.0001 vs. Adult; ^#^*P* < 0.05 vs. Old. Arrow indicates the initiation of gavaging period

Weekly BW, BT and food intake were assessed in male and female mice. In both sexes, Old groups had greater BW and lower BT compared to Adult groups throughout the experimental timeline (Figs. 1A-B, G-H, Supplemental Figure 2). No significant differences in BW or BT were observed between the PDX-treated and vehicle-treated old mice.

**Fig. 2:**
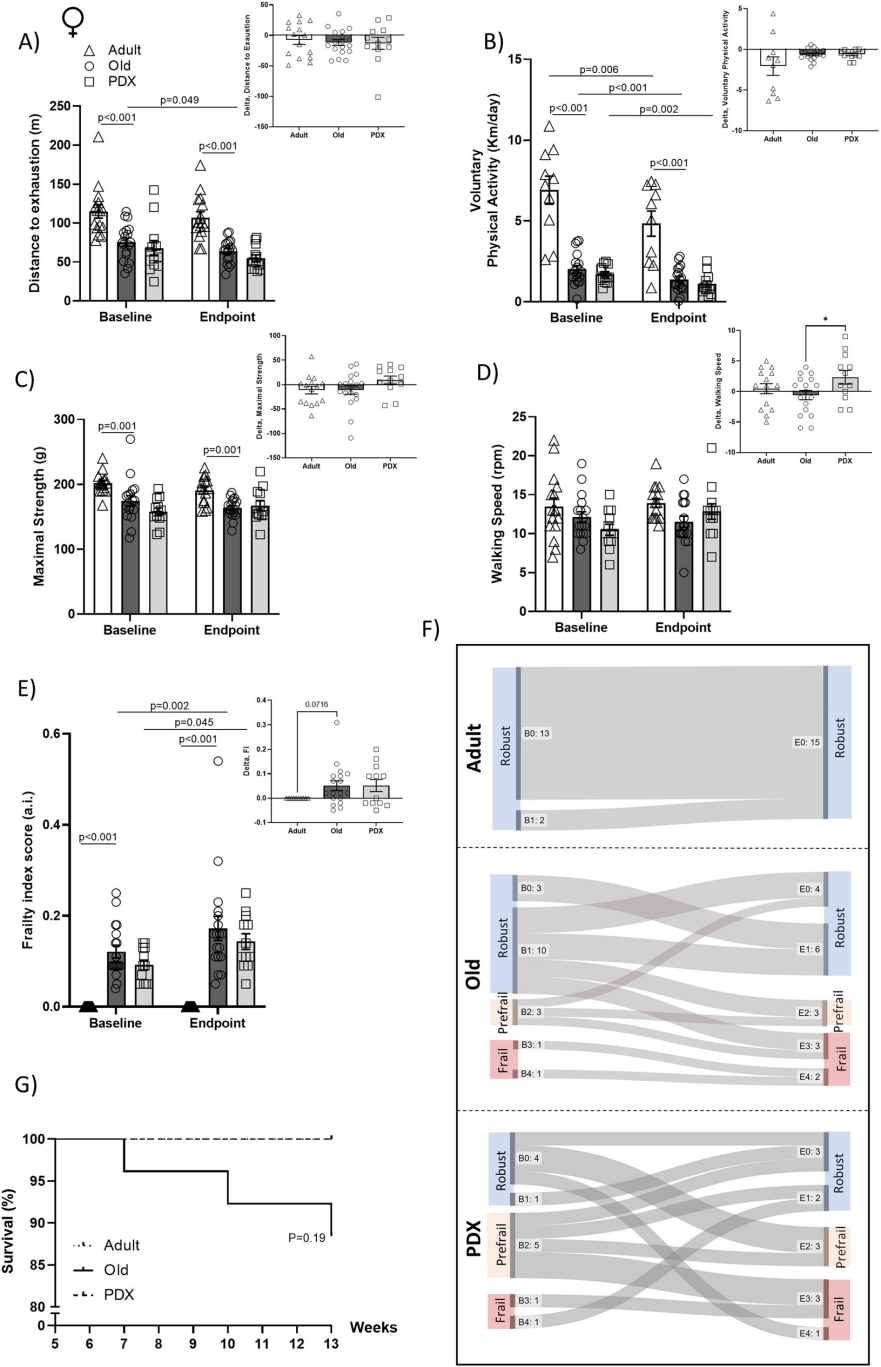
In female mice, aging is associated with decline in physical performance, which is attenuated by PDX treatment, promoting robustness. Maximal forelimb force, distance to exhaustion, voluntary wheel activity and walking speed were assessed in female mice to determine physical performance before and after PDX treatment (**A-D**, with inserted AUC). A frailty index score was determined as described in Methods at baseline and at endpoint (**E**, with AUC). Frailty index scores and performances from the physical phenotype evaluation were used to determine whether an animal was frail, prefrail or robust, as described in Methods. Sankey flow diagrams illustrate the number of animals with 0-4 health deficits at baseline (B0-B4) and at endpoint (E0-E4) and the frailty category and progression of each animal throughout the intervention period (**F**). Survival is represented as survival rate (%, **G**). Adult and Old animals were 6 months and 22 months old, respectively, at baseline, and 10 months and 26 months old, respectively, at endpoint. Data in panels A-E are expressed as mean ± SEM. Statistical analysis for all performance tests were determined by repeated measures two-way ANOVA with Bonferroni’s *post hoc* test, whereas differences in AUC values were determined by Student’s *t* test, comparing Old versus Adult and Old versus PDX. Differences in survival rate were measured by logrank (Mantel-Cox) test versus Adult. *P < 0.05 vs. Adult

During the experimental timeline, BW decreased in both Old and PDX groups, whereas an increase in BW was observed in the Adult group (both sexes) (Figs. 1A, G). In the supplement (Figs. 2A-B) we report food intake of the three experimental groups and note that the pattern of food intake is not associated with changes in BW. Table 1 highlights the weights of the visceral organs and of the fat depots between experimental groups. We observed an overall enlargement of the visceral organs, especially liver, kidney, pancreas and spleen. Lastly, based on the lower accumulation of fat (iWAT in both sexes as well as gWAT depot and BAT in males), the greater BW observed in Old vehicle-treated groups was not due to increased adiposity.

**Table 1:** Visceral organ weights in female and male mice. Results are expressed as mean ± SEM. Statistical analysis was assessed by Student's *t* test, with significant comparisons in boldface: *P < 0.05 vs. Adult within the same sex. Adult, *n*=15; Old, *n*=18; PDX, *n*=16

	**Female**			** Male**		
	Adult	Old	PDX	Adult	Old	PDX
Liver, mg/g of BW	0.04 ± 0.001	0.06 ± 0.007*	0.06 ± 0.002	0.04 ± 0.001	0.05 ± 0.002	0.05 ± 0.004
Kidney, mg/g of BW	10.15 ± 0.12	14.99 ± 0.29*	14.92 ± 0.48	10.53 ± 0.27	13.27 ± 0.4*	13.78 ± 0.4
Pancreas, mg/g of BW	7.42 ± 0.41	8.84 ± 0.56	8.81 ± 0.58	6.74 ± 0.52	8.4 ± 0.41*	9.11 ± 0.46
Spleen, mg/g of BW	3.69 ± 0.17	7.81 ± 1.72*	7.68 ± 1.09	2.27 ± 0.13	5.75 ± 1.8*	3.33 ± .040
Caecum content, g	0.37 ± 0.02	0.36 ± 0.02	0.32 ± 0.02	0.37 ± 0.01	0.41 ± 0.02	0.36 ± 0.03
gWAT, mg/g of BW	19 ± 1.5	16 ± 1.8	14 ± 1.6	24 ± 2.7	14 ± 1.4*	14 ± 0.95
iWAT, mg/g of BW	14 ± 0.64	10 ± 0.88*	11 ± 0.86	15 ± 1.4	7.2 ± 0.5*	8.2 ± 0.4
BAT, mg/g of BW	2.9 ± 0.14	2.9 ± 0.12	3.1 ± 0.18	3.0 ± 0.09	2.6 ± 0.12	2.5 ± 0.16
TA, mg/g of BW	1.6 ± 0.02	1.5 ± 0.05*	1.5 ± 0.05	1.6 ± 0.04	1.5 ± 0.05	1.6 ± 0.02
EDL, mg/g of BW	0.37 ± 0.01	0.35 ± 0.01	0.36 ± 0.01	0.34 ± 0.01	0.33 ± 0.02	0.32 ± 0.02
Soleus, mg/g of BW	0.39 ± 0.01	0.28 ± 0.01*	0.3 ± 0.01	0.38 ± 0.01	0.32 ± 0.01*	0.29 ± 0.01
Gastrocnemius, mg/g of BW	5.1 ± 0.08	3.9 ± 0.11*	3.9 ± 0.08	4.9 ± 0.15	4.1 ± 0.12*	4.1 ± 0.05

### Aging results in impaired insulin clearance in female mice

At week 13 in the experimental timeline (endpoint), animals from both sexes were submitted to an oGTT. Old vehicle-treated females had lower glycemia as compared to Adult at 120 minutes post-dextrose gavage (Fig. 1C). Despite the lower glycemic values, Old females displayed higher insulinemia both before and 120 minutes after dextrose gavage (Fig. 1D).

Plasma C-peptide concentration was determined to evaluate whether the hyperinsulinemic condition observed in the oGTT was due to alterations in pancreatic insulin secretion or due to decreased insulin clearance. We found that circulating C-peptide levels trended lower (p=0.056) in Old vehicle-treated than Adult females at 15 minutes post-dextrose gavage (Fig. 1E). Next, we calculated C-peptide/insulin ratio and noted that it was lower in the Old female group before gavage and trended lower (p=0.058) at 120 minutes post-dextrose gavage versus the Adult group (Fig. 1F), which was also validated by the calculation of the area under the curve (AUC) (Fig. 1F). Similar to females, Old vehicle-treated males had lower glycemia at baseline as compared to Adult (Fig. 1I), but no differences were noted for the insulinemia during oGTT (Fig. 1J). In both sexes, glucose homeostasis during oGTT (Figs. 1C-J) was not altered with PDX treatment.

### Age-related physical performance decline is attenuated by PDX treatment, reducing the risk of frailty

**Fig. 3:**
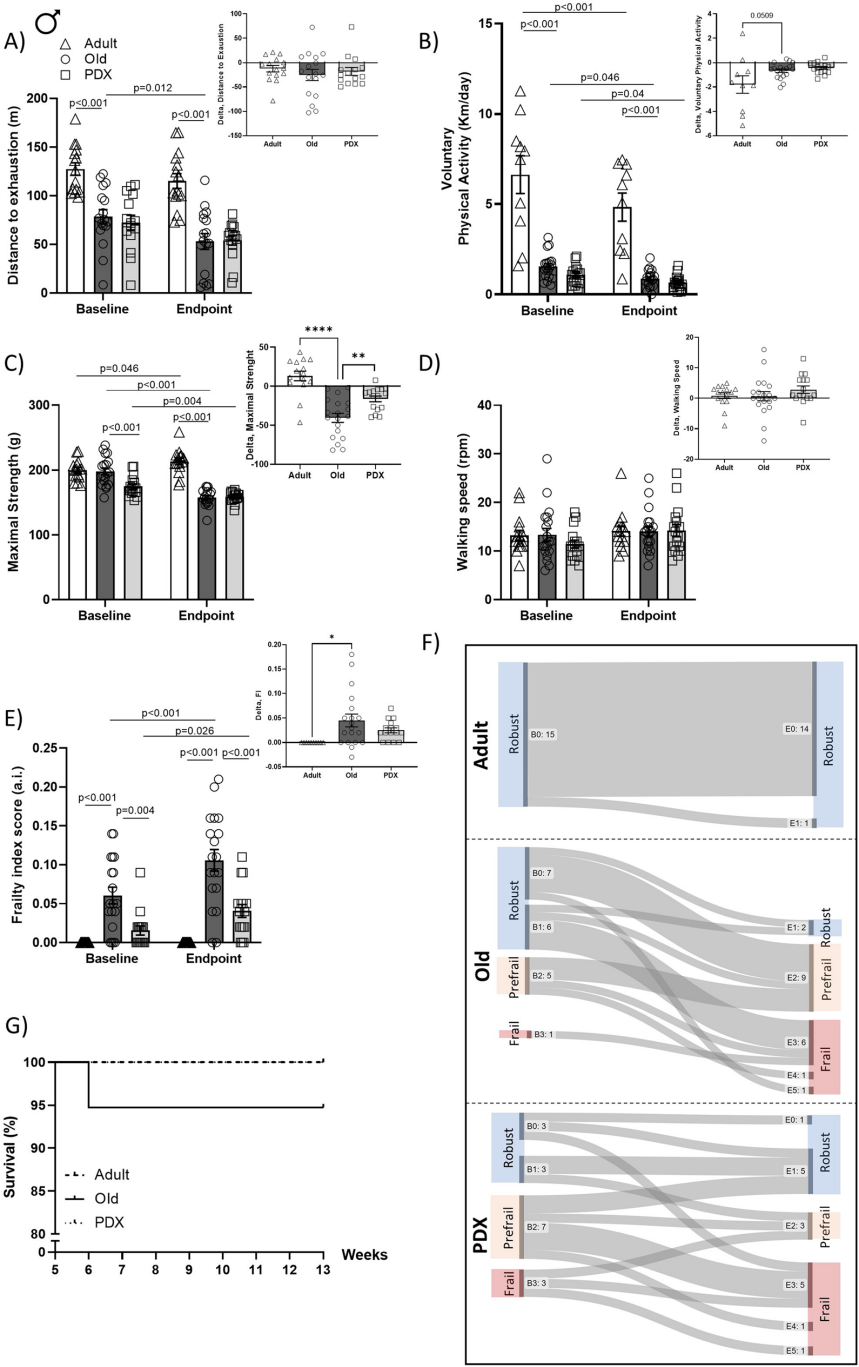
In male mice, aging is associated with decline in physical performance, which is attenuated by PDX treatment, promoting robustness. Maximal forelimb force, distance to exhaustion, voluntary wheel activity and walking speed were assessed in male mice to determine physical performance before and after PDX treatment (**A-D**, with inserted AUC). A frailty index score was determined as described in Methods at baseline and at endpoint (**E**, with AUC). Frailty index scores and performances from the physical phenotype evaluation were used to determine whether an animal was frail, prefrail or robust, as described in Methods. Sankey flow diagrams illustrate the number of animals with 0-4 positive frailty criteria at baseline (B0-B4) and at endpoint (E0-E4) and the progression of each animal throughout the intervention period (**F**). Survival is represented as survival rate (%, **G**). Adult and Old animals were 6 months and 22 months old, respectively, at baseline, and 10 months and 26 months old, respectively, at endpoint. Data in panels A-E are expressed as mean ± SEM. Statistical analysis for all performance tests were determined by repeated measures two-way ANOVA with Bonferroni’s *post hoc* test, whereas differences in AUC values were determined by Student’s *t* test, comparing Old versus Adult and Old versus PDX. Differences in survival rate were measured by logrank (Mantel-Cox) test versus Adult. *P < 0.05 vs. Adult; **P < 0.01 vs. Adult; ****P < 0.0001 vs. Adult

Frailty classification was evaluated at weeks 4 and 13 in both sexes using the combination of the physical performance (4 physical abilities), BW loss and FI. To obtain a better appreciation of physical decline, we calculated delta values for each metric (endpoint subtracted from baseline). Adult mice performed significantly better on the treadmill exhaustion test at both baseline and endpoint than Old mice (Figs. 2A, 3A). Specifically, at baseline, Old female and male mice were unable to continue running on the treadmill after reaching about 70 and 75 meters, respectively, compared to 116 and 126 meters of Adult female and male mice, respectively (Figs. 2A, 3A). These differences were maintained at endpoint. Importantly, vehicle-treated animals from both sexes showed impaired treadmill performance between baseline and endpoint, which was not statistical after PDX treatment (Figs. 2A, 3A).

Significant age-driven declines in voluntary wheel activity from baseline to endpoint were also observed in both sexes (Figs. 2B, 3B). Old vehicle-treated females and males were nearly a third less active than their Adult counterparts at both baseline and endpoint (Figs. 2B, 3B). PDX treatment did not have any effect on voluntary wheel activity.

In female mice there was a significant age-associated reduction in maximal strength at both testing periods (12.5% lower at baseline and 16% lower at endpoint) (Fig. 2C). Maximal strength in male mice was reduced by nearly 28% at the endpoint of the intervention timeline (Fig. 3C). Daily gavage with PDX attenuated the reduction in maximal strength in old male mice (Fig. 3C, delta values).

Rotarod testing was used as a measure of walking speed and as an assessment of physical coordination and learning capacities. There were no statistical differences in walking speed between the experimental groups from both sexes (Figs. 2D and 3D); however, when expressing the change in walking speed across the experimental timeline, PDX-treated females were the only group that showed a delta increase (Fig 2D).

The frailty index (FI) was calculated as the sum of scores and normalized by the 27 visually inspected health characteristics. Old mice of both sexes had higher FI scores compared to Adult mice at both baseline and the endpoint of the intervention timeline (Figs. 2E, 3E). Unexpectedly, in male animals, baseline FI scores were significantly higher (*p* = 0.004) in Old animals than PDX animals, but when comparing the change in FI score from baseline to endpoint, Old male and female mice receiving the vehicle treatment showed a similar delta in their FI score compared to the PDX-treated group (Figs. 2E, 3E, delta values). We acknowledge the disparity at baseline in the FI scores between the PDX- and vehicle-treated groups. Reasons for this disparity in FI baseline scores may arise from various factors such as the randomly assigned research design with older animals, influences with group-housing, etc. Although the observed FI differences at baseline within the experimental groups is a limitation, the statistical analyses utilized in the current study overcomes baseline differences.

Based on the combination of the 6 criteria (FI, BW loss and physical performance tests), Figs. 2F and 3F enumerate the proportion of animals classified as robust, prefrail and frail within each experimental group at each timepoint and track how the frailty status of each animal evolved during intervention. Adult male and female mice were 100% robust at baseline and at the endpoint of the intervention timeline, whereas vehicle- and PDX-treated old mice from both sexes showed a combination of mice classified as robust, prefrail, and frail at baseline. Specifically, within the Old female mice receiving vehicle treatment over the 9-week-gavaging period, 4/13 (31%) of robust animals became prefrail or frail, and 1/3 (33%) of prefrail animals became frail, whereas only one animal improved, from prefrail to robust (Fig. 2F). This decline was even more pronounced in Old vehicle-treated male mice: 13/15 (87%) of robust animals became prefrail or frail, 2/5 (40%) of prefrail animals became frail, and no animals improved (Fig. 3F). By contrast, PDX treatment for 9 weeks increased the number of animals that improved, preventing the drop in the percentage of old mice classified as robust: in PDX-treated females, although 3/5 (60%) of robust mice declined to prefrail or frail, and 2/5 (40%) of prefrail animals became frail, two prefrail animals (40%) and one frail animal (50%) improved to robust (Fig. 2F), and in PDX-treated males, while 2/6 (33%) of robust animals became prefrail or frail and 4/7 (57%) of prefrail animals became frail, 2 prefrail animals became robust (~29%) and 1 frail animal improved to prefrail (~33%).

Figures 2G and 3G show the survival curves of the mice in the study. The Adult mice (both sexes) had 100% survival during the 13-week experimental timeline. The PDX-treated old male and female mice also had 100% survival at the end of the experimental timeline (Figs. 2G, 3G) In contrast, vehicle-treated female mice had a 90% survival and the male mice had a 95% survival (Figs. 2G, 3G.)

### Aging distinctively impacts the electrical activity of the heart of female and male mice

The risk for heart failure and stroke increases with age; thus, ECG (lead II) was acquired during transthoracic echocardiography in female and male mice at weeks 4 and 13 of the experimental timeline. No differences in heart rate (HR) were noted in either sex at either time point (Tables 2, 3), but ECG analysis showed that aging was associated with prolonged QRS duration (QRS complex width) (Tables 2, 3) and alterations in specific ECG characteristics. In females, the QT interval was increased from baseline to endpoint in Old and was prolonged compared to Adult at endpoint (Table 2). We further found that, despite the random selection, QRS and QT time were longer in animals designated to receive PDX, but this difference was not seen at endpoint (Table 2.)

**Table 2:** Electrocardiogram data from female mice. Results are expressed as mean ± SEM. Statistical analysis was assessed by Student's *t* test, with significant comparisons in boldface: *P < 0.05 vs. Adult within the same time point, #*P* < 0.05 vs. Old within the same time point, Δ*P* < 0.05 vs. Baseline

Female	Adult (*n*=15)		Old (*n*=18)		PDX (*n*=12)	
	Baseline	Endpoint	Baseline	Endpoint	Baseline	Endpoint
HR (bpm)	333 ± 59	367 ± 65	339 ± 84	329 ± 78	346 ± 82	354 ± 106
QRS (msec)	12.6 ± 1.6	13.0 ± 0.9	13.6 ± 1.3*	14.2 ± 1.0*	**14.7 ± 0.8**^**#**^	14.2 ± 1.4
QT (msec)	27.0 ± 5.8	27.2 ± 3.5	27.2 ± 4.1	30.9 ± 5.1*^Δ^	**30.8 ± 3.8**^**#**^	28.9 ± 4.3
PR (msec)	43.9 ± 8.3	41.7 ± 4.1	47.3 ± 6.2	45.4 ± 7.8	43.5 ± 6.2	46.1 ± 6.7
T wave duration (msec)	8.1 ± 3.3	7.0 ± 3.4	9.6 ± 2.9	9.7 ± 5.3	8.4 ± 2.9	8.4 ± 4.1
P wave duration (msec)	13.7 ± 5.2	11.8 ± 2.6	15.0 ± 7.2	11.8 ± 4.2	11.6 ± 2.2	14.5 ± 8.7
Amp P (mV)	65.3 ± 30.8	49.2 ± 13.2	58.3 ± 16.6	71.8 ± 42.0*	69.0 ± 21.2	85.6 ± 53.7
Amp Q (mV)	-65.9 ± 21.6	-51.4 ± 27.2	-44.8 ± 37.6	**-70.6 ± 54.0**^**Δ**^	-49.4 ± 21.2	-74.4 ± 64.3
Amp R (mV)	445.4 ± 136.5	561.8 ± 132.8	339.8 ± 203.6	388.3 ± 214.6*	344.9 ± 169.9	347.6 ± 182.4
Amp S (mV)	-120.4 ± 88.3	-107.7 ± 79.0	-65.0 ± 59.4	-84.8 ± 72.3	-74.7 ± 117.6	-96.6 ± 133.7

**Table 3:** Electrocardiogram data from male mice. Results are expressed as mean ± SEM. Statistical analysis was assessed by Student's *t* test, with significant comparisons in boldface: *P < 0.05 vs. Adult within the same time point, #*P* < 0.05 vs. Old within the same time point, Δ*P* < 0.05 vs. Baseline

Male	Adult (*n*=15)		Old (*n*=18)		PDX (*n*=12)	
	Baseline	Endpoint	Baseline	Endpoint	Baseline	Endpoint
HR (bpm)	343 ± 50	294 ± 51	329 ± 58	343 ± 85	356 ± 108	378 ± 88
QRS (msec)	13.1 ± 1.5	13.9 ± 0.9	14.7 ± 1.8*	14.5 ± 0.8	15.0 ± 1.4	14.6 ± 1.3
QT (msec)	26.1 ± 4.8	**30.1 ± 2.6**^**Δ**^	28.9 ± 6.0	28.0 ± 4.8	28.7 ± 5.0	27.9 ± 4.6
PR (msec)	46.4 ± 8.4	**40.1 ± 7.0**^**Δ**^	47.4 ± 7.0	**41.2 ± 8.5**^**Δ**^	45.0 ± 9.6	**47.8 ± 9.9**^**#**^
T wave duration (msec)	5.2 ± 4.9	5.3 ± 1.6	8.9 ± 2.2*	8.1 ± 3.5*	10.0 ± 3.6	8.2 ± 3.6
P wave duration (msec)	17.5 ± 13.7	**11.3 ± 2.9**^**Δ**^	11.6 ± 3.6*	10.3 ± 2.3	10.1 ± 3.3	10.8 ± 4.4
Amp P (mV)	61.8 ± 21.9	70.5 ± 13.2	66.0 ± 33.4	42.9 ± 14.9*^Δ^	61.4 ± 47.0	**40.4 ± 21.8**^**Δ**^
Amp Q (mV)	-42.5 ± 21.4	-63.5 ± 34.4	-74.2 ± 72.9	**-45.3 ± 39.6**^**Δ**^	-59.6 ± 33.7	**-33.5 ± 16.4**^**Δ**^
Amp R (mV)	483.9 ± 124.5	**544.6 ± 137.9**^**Δ**^	320.2 ± 142.0*	342.5 ± 161.5*	376.4 ± 150.3	388.5 ± 174.9
Amp S (mV)	-116.4 ± 85.8	**-164.9 ± 76.0**^**Δ**^	-23.1 ± 52.3*	-20.1 ± 44.3*	-27.3 ± 66.9	-29.4 ± 80.7

Similarly, male vehicle-treated Old mice had prolonged QRS at baseline when compared to Adult (Table 3.) In females no differences were observed in PR, T duration or P duration (Table 2.) In males, the PR duration was decreased overtime in Adult and Old, which was not observed in PDX-treated, resulting in longer PR time at endpoint as compared to vehicle-treated (Table 3.) Furthermore, Old male had longer T and P duration versus Adult (Table 3.)

In females, Amp P was enhanced by age, whereas Amp R was decreased as compared to Adult at endpoint (Table 2.) By contrast, in males, we observed a shortening of the Amp P overtime in both Old and PDX groups, resulting in lower Amp P at endpoint in Old as compared to Adult (Table 3.) As for the females, Amp R was lower in Old versus Adult (Table 3) and Amp S was reduced with aging in both sexes, although it was only statistically significant in males (Tables 2, 3.)

### Aging is associated with mesangial expansion and glomerular hypertrophy, but not muscle atrophy

Because we observed age-associated enlargement of the kidney in this study (Table 1, Supplemental Figure 3A), and chronic kidney disease is prevalent among older adults and is associated with a decline in glomerular filtration rate and impairments of renal structure [32], we evaluated glomerular structure in H&E-stained kidney cortex preparations from female mice. This analysis confirms that aging is associated with glomerular enlargement (Supplemental Figure 3C) and mesangial expansion (Supplemental Figure 3D). Although PDX treatment have previously attenuated kidney failure in end-stage renal disease [19], it did not alter kidney structure in the current study (Supplemental Figures 3A-D.)

We did not observe muscle atrophy between 6-22 months in female mice (Supplemental Figure 3E-G). In a subset of animals analyzed for single fiber CSA, we detected a hypertrophic-trend in the PDX-treated old females (increase CSA of EDL) when compared to vehicle-treated (Supplemental Figure 3E-F). Although there is a hypertrophic-trend in single fiber CSA of the EDL, the wet weight of the soleus and EDL did not change between experimental groups.

### Aging upregulates the production of circulating inflammatory cytokines

Because chronic inflammation is one of the most important biological processes involved in aging, we measured the level of several cytokines in plasma, and performed comparisons between animals of the same sex. In both sexes, circulating MCP-1 was significantly elevated (*p* < 0.05) in Old animals compared to Adult animals; similarly, IL-6 and TNF-α levels were substantially increased in Old animals of both sexes, although this increase was significant only in male animals (Supplemental Table 1). PDX treatment showed a trend to attenuate the increase in circulating MCP-1 in female animals (*p* = 0.076.)

### Aging produces numerous transcriptional changes in the liver, a subset of which are partially reversed by PDX treatment

We sequenced RNA extracted from the liver of 15 female mice, five from each experimental group, in order to assess the transcriptional consequences of aging and PDX treatment. The data were of high quality (Supplemental Table 2), with robust sequencing depth (36.0 ± 4.4 million read pairs, mean ± s.d.) and overall alignment rate (95.7% ± 0.8%) and relatively low amounts of mitochondrial RNA (3.6% ± 0.5%). Of the non-mitochondrial read pairs that aligned uniquely to the genome in proper pairs, the vast majority (94.1% ± 0.6%) were assigned to a single Ensembl Gene locus.

Principal Component Analysis (PCA) was used to cluster samples with respect to the expression of all genes in the transcriptome (Supplemental Figure 4). In this analysis, the Adult group clearly separated from both the Old and PDX mice, whereas the Old and PDX groups did not separate from each other, indicating that aging resulted in more pronounced changes in gene expression than PDX treatment. Moreover, there was a large amount of variability in the Old group, with strong changes in gene expression between animals O2 and O5 and the remaining animals (i.e., separation with respect to Principal Component 1, which explains 22% of total variance in the experiment.) Differential gene expression was then assessed between pairs of experimental groups, and in accordance with the clustering by PCA, there was strong differential expression between the Adult and Old groups (1,621 genes at FDR *q* < 0.05) but no significant gene-level changes between the Old and PDX groups (0 genes at FDR *q* < 0.25) (Supplemental Table 3).

**Fig. 4:**
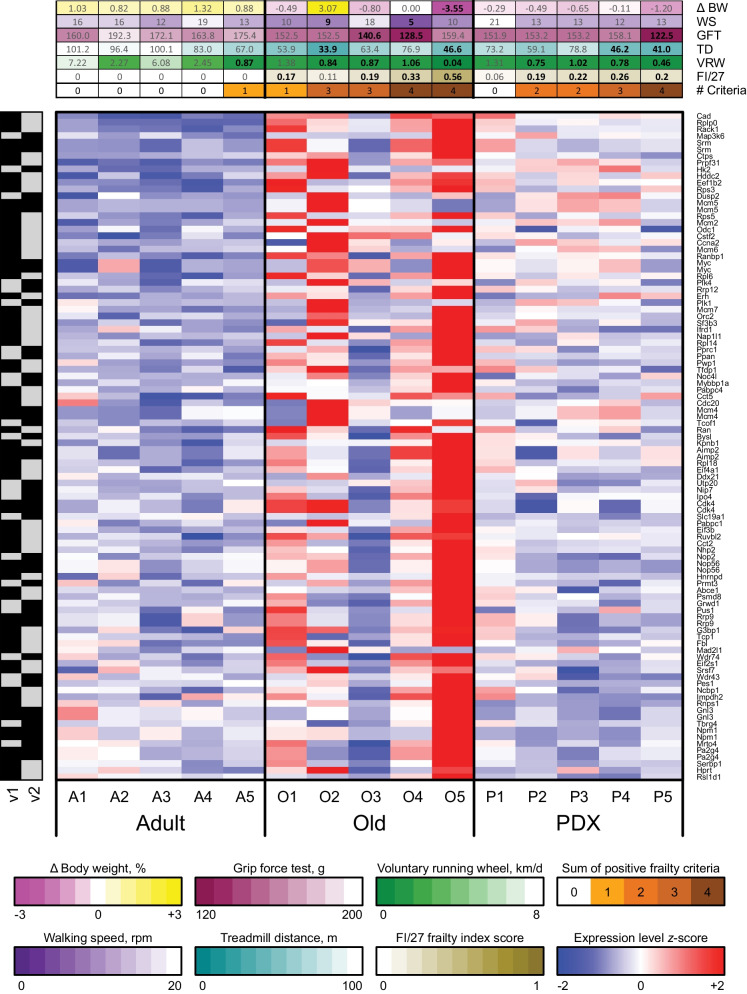
Targets of the transcription factor Myc are coordinately upregulated in the liver of female animals with aging, which is attenuated by PDX treatment. The heatmap indicates the variance-stabilizing-transformed (VST) expression of the union set of genes that are in the leading edges of the two Hallmark Myc target gene sets (v1 and v2) for both comparisons (coordinately downregulated in Old vs Adult, FDR *q* < 0.05; coordinately upregulated in PDX vs Old, FDR *q* < 0.05). Rows and columns correspond to genes and samples, respectively, and the presence or absence of each gene within each gene set is indicated by gray and black boxes, respectively. Blue and red indicate VST expression values that are at least 2 standard deviations below or above, respectively, the mean value (white) of each row. The various frailty metrics are shown in sidebars above each column: average weekly percent change in body weight over the 12-week period of the study (Δ BW), and walking speed (WS), grip force test (GFT), treadmill distance (TD), voluntary running wheel activity (VRW), and FI/27 frailty index score measured at endpoint. The bottommost column sidebar indicates the tally of positive frailty criteria, i.e., the number of metrics that fall within the frailest quintile across all animals in the study (indicated in bold face). Color scales are shown below the heatmap, with darker colors corresponding to greater frailty. Rows are sorted from top to bottom by Old vs Adult Wald statistic, and columns are sorted from left to right first by sample group, then by number of positive frailty criteria, and finally by FI/27 frailty index score

**Fig. 5:**
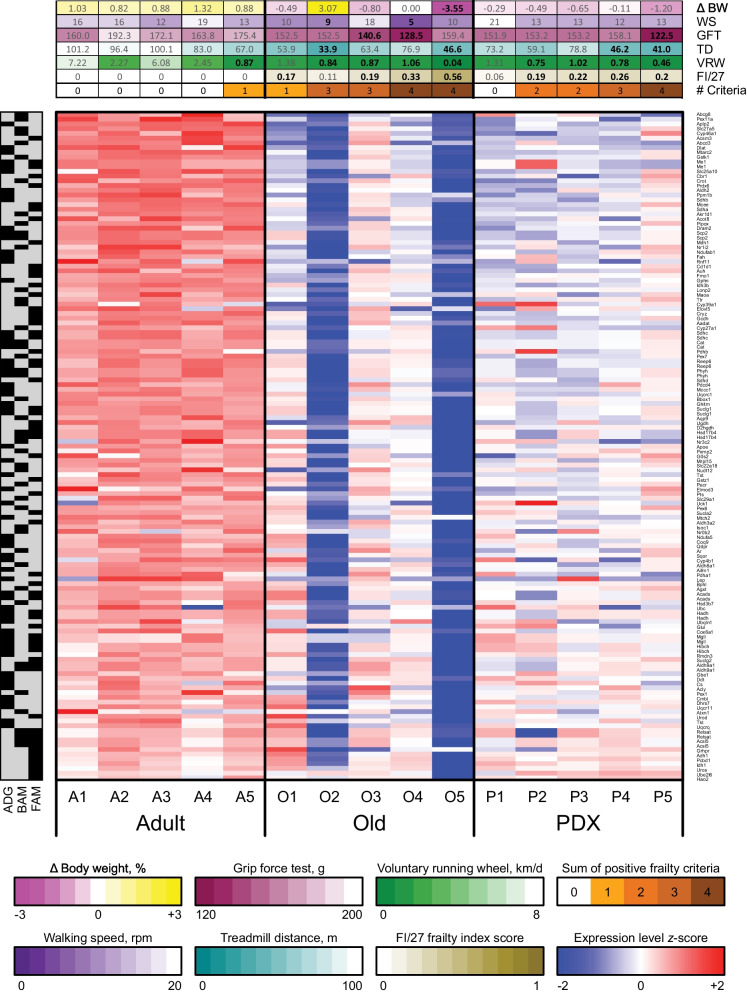
Genes involved in lipid metabolism are coordinately downregulated in the liver of female animals with aging, which is partly attenuated by PDX treatment. The heatmap indicates the variance-stabilizing-transformed (VST) expression of the union set of genes that are in the leading edges of the Hallmark adipogenesis (ADG), bile acid metabolism (BAM), and fatty acid metabolism (FAM) gene sets for both comparisons (coordinately downregulated in Old vs Adult, FDR *q* < 0.05; coordinately upregulated in PDX vs Old, FDR *q* < 0.05). Rows and columns correspond to genes and samples, respectively, and the presence or absence of each gene within each gene set is indicated by gray and black boxes, respectively. Blue and red indicate VST expression values that are at least 2 standard deviations below or above, respectively, the mean value (white) of each row. The various frailty metrics are shown in sidebars above each column: average weekly percent change in body weight over the 12-week period of the study (Δ BW), and walking speed (WS), grip force test (GFT), treadmill distance (TD), voluntary running wheel activity (VRW), and FI/27 frailty index score measured at endpoint. The bottommost column sidebar indicates the tally of positive frailty criteria, i.e., the number of metrics that fall within the frailest quintile across all animals in the study (indicated in bold face). Color scales are shown below the heatmap, with darker colors corresponding to greater frailty. Rows are sorted from top to bottom by Old vs Adult Wald statistic, and columns are sorted from left to right first by sample group, then by number of positive frailty criteria, and finally by FI/27 frailty index score

**Fig. 6:**
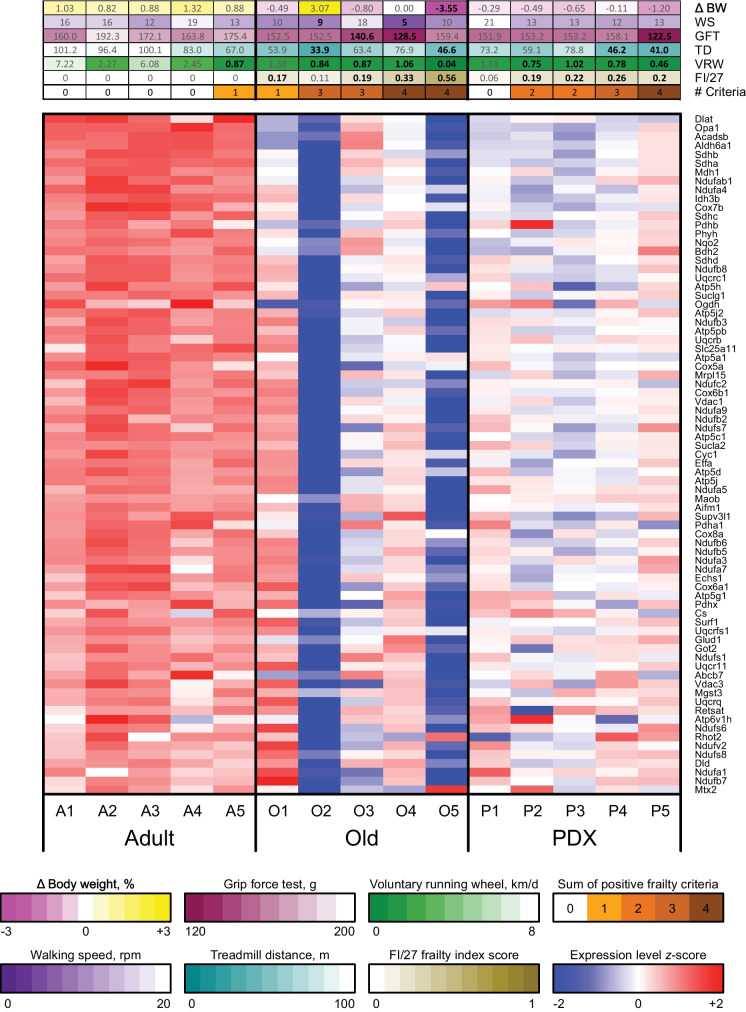
Genes involved in oxidative phosphorylation are coordinately downregulated in the liver of female animals with aging, which is partly attenuated by PDX treatment. The heatmap indicates the variance-stabilizing-transformed (VST) expression of the genes that are in the leading edges of the Hallmark oxidative phosphorylation gene set for both comparisons (coordinately downregulated in Old vs Adult, FDR *q* < 0.0001; coordinately upregulated in PDX vs Old, FDR *q* < 0.0001). Rows and columns correspond to genes and samples, respectively. Blue and red indicate VST expression values that are at least 2 standard deviations below or above, respectively, the mean value (white) of each row. The various frailty metrics are shown in sidebars above each column: average weekly percent change in body weight over the 12-week period of the study (Δ BW), and walking speed (WS), grip force test (GFT), treadmill distance (TD), voluntary running wheel activity (VRW), and FI/27 frailty index score measured at endpoint. The bottommost column sidebar indicates the tally of positive frailty criteria, i.e., the number of metrics that fall within the frailest quintile across all animals in the study (indicated in bold face). Color scales are shown below the heatmap, with darker colors corresponding to greater frailty. Rows are sorted from top to bottom by Old vs Adult Wald statistic, and columns are sorted from left to right first by sample group, then by number of positive frailty criteria, and finally by FI/27 frailty index score

Gene Set Enrichment Analysis (GSEA) was then performed to assess the coordinate regulation of 50 gene sets representing well-characterized ("Hallmark") pathways, obtained from the Molecular Signatures Database (MSigDB) (Supplemental Table 4 and Supplemental Table 5). Numerous gene sets were significantly (FDR *q* < 0.05) coordinately upregulated with respect to aging (Old versus Adult), including those representing several inflammatory pathways (IL-6/JAK/STAT3 signaling, inflammatory response, TNFα signaling via NFkB), Myc signaling, apoptosis, and hypoxia, whereas those related to oxidative phosphorylation, bile acid and fatty acid metabolism, and adipogenesis were significantly coordinately downregulated. Interestingly, many of these pathways were significantly (FDR *q* < 0.05) coordinately regulated in the opposite direction with respect to PDX treatment (PDX versus Old), including Myc targets, adipogenesis, bile acid and fatty acid metabolism and oxidative phosphorylation. The expression of the "leading edge" genes (i.e., those that contributed the most to the pattern of coordinate regulation) from several of these gene sets is shown in Figs. 4, 5, and 6.

### PDX treatment prevents age-driven loss of bone mineral density

**Fig. 7:**
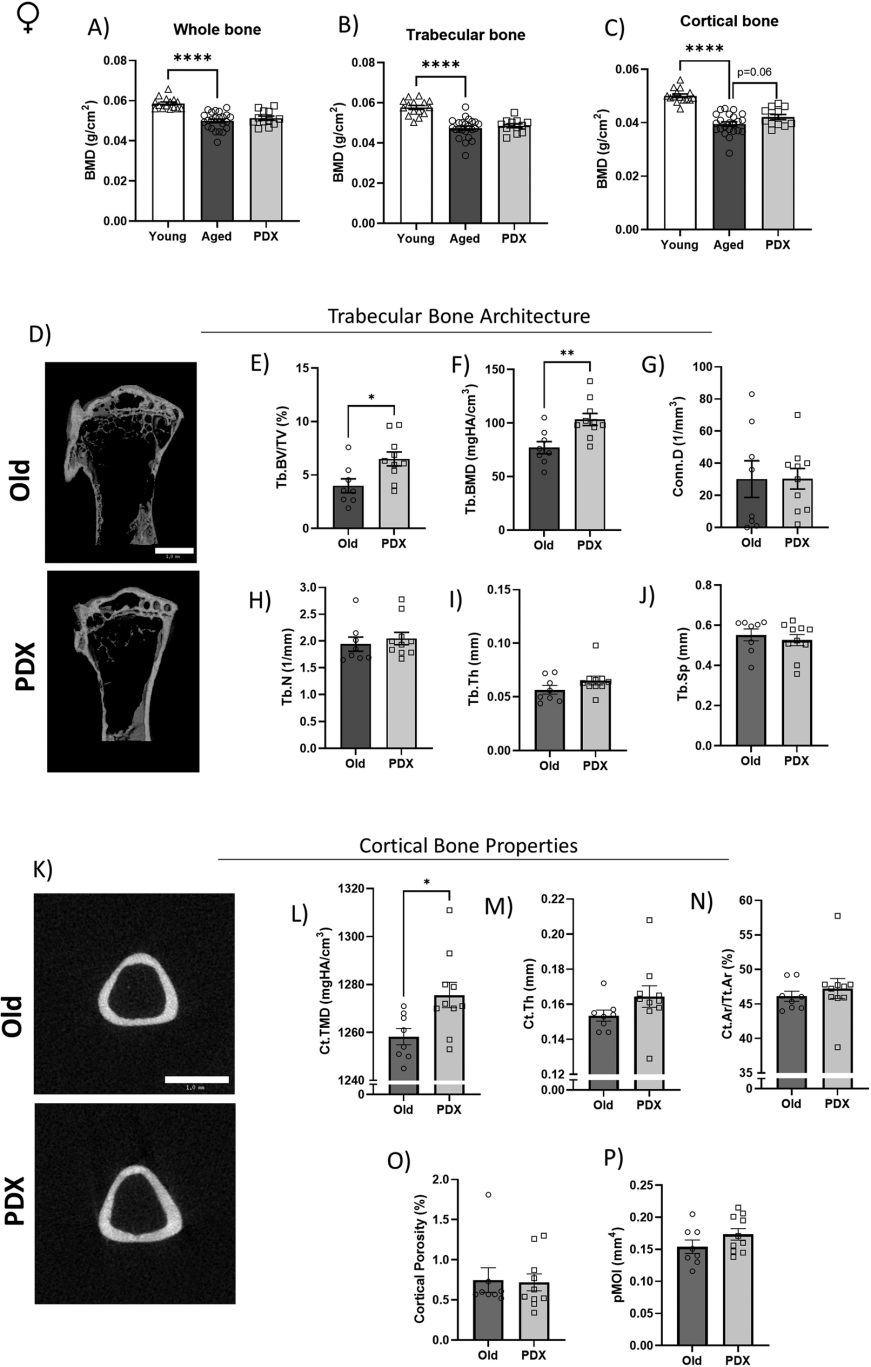
Age-driven decline in bone mineral density is attenuated by PDX treatment in female mice. Old vehicle-treated and PDX-treated female mice were euthanized at 25 months of age and tibias were isolated and fixed in 4% formaldehyde. Bone mineral density (BMD) from trabecular (**A**), cortical (**B**) and whole bone (**C**) was analyzed by dual energy X-ray absorptiometry (DEXA). 3D bone morphology was further analyzed in tibia from Old vehicle-treated and PDX-treated animals using Micro-CT. Trabecular bone architecture (**D**) is illustrated by BV/TV (**E**), BMD (**F**), Conn.D (**G**), Tb.N (**H**), Tb.Th (**I**) and Tb.Sp (**J**); whereas cortical bone properties (**K**) are shown by Ct.TMD (**L**), Ct.Th (**M**), Ct.Ar/Tt.Ar (**N**), cortical porosity (**O**) and pMOI (**P**). Data are expressed as mean ± SEM. All *P* values were determined by Student’s *t* test comparing Old versus PDX. *P < 0.05 vs. Adult; **P < 0.01 vs. Adult; ****P < 0.0001 vs. Adult. BMD: Bone mineral density; BV/TV: Bone Volume Fraction; Conn.D: Connectivity Density; Tb.N: Trabecular Number; Tb.Th: Trabecular thickness; Tb.Sp: Trabecular Separation; Ct.TMD: Cortical Tissue Mineral Density; Ct.Th: Cortical Thickness; Ct.Ar/Tt.Ar: Bone Area Fraction; pMOI: Polar Moment of Inertia

To gain insight into bone health, we assessed the bone mineral density (BMD) of the tibia harvested from female and male mice. DEXA analysis showed an age-related decline in BMD as revealed by lower whole, trabecular and cortical bone mineral density values in females (Figs. 7A-C). In males, only the cortical bone mineral density was lower as compared to Adult (Supplemental Figure 5C).

Because we observed that the PDX-treated old female group trended (p=0.06) to a greater cortical bone mineral density compared to the vehicle-treated Old group (Fig. 7C), we performed microCT analysis of Old-vehicle versus PDX-treatment female mice. Trabecular bone architecture data confirmed that PDX treatment prevented loss of bone volume (Tb.BT/TV) and mineral density (Tb.BMD) compared to vehicle-treated animals (Figs. 7D-F), whereas connective density (Conn.D), trabecular number (Tb.N), thickness (Tb.Th) and separation (Tb.sp) were not statistically different between groups (Figs 7G-J). In the cortical region, PDX prevented age-driven loss in cortical tissue mineral density (Ct.TMD) (Figs. 7K, L), while there were no statistically significant changes in cortical thickness (Ct.Th), bone area fraction (Ct.Ar/Tt.Ar), cortical porosity or polar moment of inertia (pMOI) (Figs. 7M-P).

## Discussion

Aging is characterized by a progressive loss of organ function and resilience. Herein we aimed to investigate the therapeutic potential of PDX to attenuate aging-driven impairments. We further investigate, in a multi-organ perspective, age-related declines in male and female mice from 6 months (Adult baseline, equivalent to 20-30 years in humans) to 10 months (Adult endpoint, 38–47 years in humans) and from 22 months (Old baseline, 56–69 years in humans) to 25 months (Old endpoint, ≥ 70 years in humans) [33]. Resilience declines over time during human lifespan, facilitating the onset of frailty. In healthy aging, frailty is expected to be prevalent only in adults above 65 years of age, specifically in those of middle-old age (75-84 years) [34]. However, early frailty onset might be observed in adults with poor lifestyle (low physical activity, smoking), unfavorable social demographic factors (low education, loneliness) and/or chronic diseases (obesity, diabetes) [34]. In our cohort, using genetically homogeneous animals and controlled housing and diet, the onset of frailty was only influenced by advanced age (demographic factor) and low physical activity (lifestyle factor). As we expected a much severer decline in physical performance from 22 to 25 months old (late middle age to older adulthood) than from 6 to 10 months old (adulthood to middle age), the 16-month age gap between Adult and Old animals in this study allowed us to best address the physical and biological impairments that occur from late middle age to older age.

### Frailty

Many studies use treadmill, grip force test, activity cages and rotarod to identify overall physical frailty status, but this is the first time that those physical performance tests are merged with the frailty index (FI) score and BW to define the frailty status of mice. We observed that, applied individually, these frailty assessment methods would classify distinct subsets of animals from the same cohort as frail (data not shown), indicating that they are rather complementary (also proposed by others [35]). Similarly, a study with adults aged 50+ from the National Health and Nutrition Examination Survey identified the prevalence of frailty as 3.6% using the modified frailty phenotype and 34% using the frailty index [36], reinforcing their disparities. Thus, we believe that the combination of these tools enriches the classification of frailty, integrating multiple physical manifestations of systemic decline.

Poor mobility not only decreases the quality of daily life, but also increases the risk and severity of falls, and therapies to combat physical decline are urgently needed. In the current study, we found several positive outcomes with PDX treatment that were sex-specific: preservation of endurance performance was observed in both sexes, improved walking speed was observed in females, and alleviation of strength loss was observed in males. This is the first time that PDX has been described as a therapy against physical decline in aging. We further demonstrate that PDX treatment promoted robustness during the 3-month experimental protocol in both sexes, while vehicle-treated animals had a dramatic loss of animals classified as robust, especially in males.

PDX is a DHA-derived specialized pro-resolving lipid mediator (SPM) that acts in the resolving phase of inflammation in both acute and chronic inflammatory diseases [37]. Aging is associated with a decline in the levels of SPMs, such as lipoxin A4, resolvin D6, protectin D1/DX and maresin 1, and an attendant increase in inflammation and delay in recovery of skeletal muscle strength [38]. We therefore believe that combating this biosynthetic deficiency would potentially delay the onset of frailty. Indeed, fish oil therapy slowed the normal decline in muscle mass and function in older adults [39] and in hemodialysis patients [40]. In patients with non-small-cell lung cancer, daily consumption of fish oil maintained muscle mass during chemotherapy [41]. High circulating levels of omega-3 PUFAs in plasma prevented a decrease in gait speed in women [42] and in community-dwelling older adults [43].

### Bone

Trabecular bone mass and cortical thickness decrease with aging [44], which is more prominent in women [45, 46]. Aged individuals have a decline in the osteoblast:osteoclast ratio, favoring bone mass loss [47] and increased bone fragility, leading to increased risk of fractures and subsequent loss of mobility [48]. Given the dramatic increase in the age of world’s population, the consequences of osteoporosis represent a huge burden to public health [48], and therapies to combat osteoporosis are therefore needed. FDA-approved therapies against osteoporosis include bisphosphonates, which bind to the bone mineral surface and inhibit osteoclast-mediated bone resorption [49], and teriparatide (the amino terminal region of PTH) which acts as an anabolic agent by promoting new bone formation and, reducing the risk of vertebral and hip fractures [50, 51]. Novel therapeutic strategies, including neutralizing antibodies against sclerostin (Romosozumab), RANK ligand (Denosumab) and PTHrP analog (Abaloparatide), have an additional anti-osteoporotic effect [48]; however, these interventions also cause adverse effects, highlighting the need for alternative treatment [52]. In the present study, the most appealing therapeutic effect of PDX centers on preservation of bone mineral density. This is the first time that the beneficial effect of PDX on preventing loss of bone mineral density is reported.

### Liver transcriptomics

As a major orchestrator of metabolic processes, the liver plays a central role in the development of frailty. There are well-characterized age-associated changes in the morphology and volume of liver cells, associated with the accumulation of lipofuscin (granules of damaged, undegraded protein), which in turn increases the generation of reactive oxygen species (ROS) [53]. The aged liver is also marked by genomic and epigenomic alterations that contribute to dysregulation of mitochondrial function and nutrient sensing pathways, leading to cellular senescence and low-grade inflammation [54]. For these reasons, changes in liver transcriptomic signatures have been used as predictors of age-related degenerative processes and frailty in mice [55, 56]. We therefore decided to investigate the hepatic transcriptomic profile of vehicle- and PDX-treated old female mice (to our knowledge, the first study of its kind) to identify whether our aging model would trigger changes in liver gene expression and whether PDX treatment would attenuate or reverse any of the observed alterations.

In this study, a set of inflammatory response genes and expression signatures of IL-6/JAK/STAT3 and TNF-α signaling were coordinately upregulated in Old animals compared to Adult animals. These gene sets include *Ccl2*, *Il6*, and *Tnf*, which are consistent with the overall pattern of upregulation of the corresponding cytokines MCP-1, IL-6 and TNF-α in the plasma of female animals. We also observed that targets of Myc were coordinately upregulated with respect to aging, and that this upregulation was largely attenuated by PDX treatment. Myc is a highly pleiotropic transcription factor that coordinates many intracellular and extracellular programs required for tissue growth and repair [57], and failure of its negative feedback transcriptional autoregulation leads to its accumulation [58, 59]. There is substantial evidence that deregulation in Myc-mediated cell proliferation may promote cancer [57, 60, 61] and the complex biological decline associated with aging increases the odds of cancer [62]. Thus, understanding Myc regulation may be indicated not only as a therapeutic opportunity to counter tumor growth [59], but also frailty. Early studies of the transcriptional regulation of Myc in the context of aging were inconclusive: in 1987, Matocha et al. observed that Myc transcription was strongly elevated in the liver of 22-month-old Fischer-344 rats compared to 4-month-old rats [63], whereas in 1989, using C57BL/6NJcl mice, Ono et al reported a drastic decrease in the amount of liver Myc mRNA in middle-aged (14 months) and old (26 months) animals relative to young (2 months) animals [64]. However, in a more recent study in *Drosophila*, overexpression of Myc was found to shorten adult lifespan, and conversely, Myc haploinsufficiency led to extended lifespan [65]. Similarly, in C57BL/6 mice, Hofmann and colleagues showed that Myc^+/-^ animals had increased longevity relative to Myc^+/+^ animals, as well as an improved healthspan, reflected by reductions in cardiac fibrosis, bone density loss and hepatic lipid droplet size and improved performance by rotarod test [60]. Herein, we observed that Myc targets were down-regulated with PDX treatment, which paralleled some improvements in physical performance and bone mineral density loss in females. Further investigation is needed to elucidate whether PDX-driven therapeutic benefits are mediated by Myc targets.

The aging process is marked by the progressive depletion of NAD^+^, a coenzyme that regulates cellular bioenergetics and adaptive stress responses and whose interconversion to and from its reduced form, NADH, is essential for oxidative phosphorylation (oxphos) [66]. It is also known that aging is associated with the reduced production of mitochondrial enzymes, such as mitochondrial nitric oxide synthase, manganese superoxide dismutase (MnSOD), and complexes I and IV [67]. Accordingly, in our study, an expression signature of oxphos, including genes encoding subunits of NADH:ubiquinone oxidoreductase (Complex I), is coordinately downregulated in Old animals compared to Adult animals. Conversely, restoring NAD^+^ concentration has been shown to be beneficial against impaired aging in rodents [68–71]. This may be accomplished through dietary administration of SPMs; for example, the consumption of DHA and EPA for 3 weeks increased NAD^+^ and its related metabolites in the aortic arch of pro-atherosclerotic apolipoprotein E-deficient (ApoE^−/−^) mice [72]. The expression of the oxphos signature is coordinately upregulated by PDX treatment relative to Old vehicle-treated animals, indicating that therapeutic/nutraceutical strategies might be successful in conserving NAD^+^ function in later life.

It is important to note that there was a large amount of variability in gene expression within the Old animals; specifically, the difference between animals O2 and O5 and the remainder of the mice explained roughly 20% of the total variance in the experiment. The reason for this disparity did not appear to be technical, as the alignment metrics of these two samples were similar to those of the other samples; rather, it appears that these two animals fall at the extreme end of the frailty spectrum, representing between them the highest FI score and average weekly percent weight loss and the lowest treadmill distance to exhaustion and voluntary running wheel activity across all animals in the study at endpoint. This complicates the interpretation of the RNA-seq data, as these two samples drove much of the significant differential regulation of pathways between the Old animals and the Adult or PDX groups. However, as the onset of frailty is not expected to occur in a synchronous or homogeneous manner. For example, these two animals were identified as frail (O2) and robust (O5) at baseline. Thus, the variability in gene expression is not surprising; rather, it underscores the need for further studies to fully elucidate the specific effects of aging and PDX treatment on hepatic gene expression.

### Insulin clearance

We found several areas of agreement between physiologic metrics of insulin signaling and liver transcriptomic data. Old mice had an overall reduction of adipose tissue weight relative to Adult animals, which corresponded with the coordinate downregulation of genes associated with fatty acid metabolism and adipogenesis. Furthermore, during oGTT, we found that aging was associated with hyperinsulinemia in female mice, which was not triggered by hyperglycemia or enhanced insulin secretion, but rather by decreased insulin clearance. Indeed, aging results in the defenestration of liver sinusoidal endothelial cells [73], which in turn may impair insulin clearance, resulting in hyperinsulinemia and hepatic insulin resistance [74]. Similar to our findings, Marmentini C. and collaborators showed age-driven reduction in insulin clearance when comparing 3- and 18-month-old mice, which was associated with the downregulation of carcinoembryonic antigen-related cell adhesion molecule-1 (CEACAM1) and insulin-degrading enzyme (IDE) levels and activity [75]. In our study, *Ceacam1* was significantly downregulated in Old versus Adult animals (*p* = 0.006, FDR *q* = 0.06), whereas *Ide* was significantly upregulated (*p* = 2x10^-5^, FDR *q* = 0.002), which could potentially explain changes in insulin clearance.

### Body weight

During the course of the experiment, we observed age-, but not PDX-specific changes in BW: Old mice progressively lost weight, likely due to loss of both fat and lean mass, whereas Adult mice gained weight. Importantly, fluctuations in BW were not related to food intake, indicating that other physiological processes were likely involved. There are many health benefits associated with weight loss; however, it is also frequently observed among frail individuals [76], and unintentional weight loss is associated with higher mortality rate in older adults [77–79]. The weight loss observed in Old mice in this study is consistent with the Fried unintentional weight loss criterion for frailty in humans [3] as well as other groups that found BW loss as a positive criteria for frailty in mice [80]. Because the Adult mice gained weight during our experiment, it is logical to assume that the Old mice had also gained weight earlier in their lifespan, but at some point, started losing fat and lean mass, as reported in older humans (65+ years old) [81, 82]. Moreover, our research team has previously shown that, despite losing weight at the edge of lifespan, mice that were heavier near the midpoint of their lifespan died earlier [10]. Taken together, these findings suggest that body weight alterations throughout the lifespan is not a straight-forward process.

### Kidney

Our findings also demonstrated that kidney enlargement in old females was likely due to glomerular hypertrophy and mesangial expansion. Mechanistically, age-driven mesangial expansion correlated with impaired laminin α1 (LAMA 1) in regulating mesangial cell behavior via TGF-β/Smad signaling [83]. Indeed, renal aging correlates with impaired renal structure and function [84]. Yet, in contrast with our findings, kidney weight was previously shown to progressively decline by 20–30% in humans between 70 and 90 years of age [85]. In a previous study, PDX treatment largely protected kidney function and structure against end-stage-renal-disease [19], but we did not observe the same in our aging mouse model. These discrepancies are likely due to the different models and biological ages explored.

### Muscle mass and CSA

In this study, we observed an age-associated overall reduction of skeletal muscle mass, which is consistent with the published literature focused on sarcopenia [86–88]. There are many cellular mechanisms that contribute to a loss of muscle mass, such as alterations in protein homeostasis that result in single fiber atrophy [89]. In the current study, the mean CSA of the single fibers in the EDL and soleus muscles were similar between the Adult and Old groups. Similarly, a study using 12- to 22-month-old mice also did not observe age-driven atrophy in the EDL [90]. In contrast, single fiber atrophy was reported in 78-week-old (19.5-month-old) female mice [86]. The variability observed across studies is likely due to many factors, including the age of the mice or the age-span of the mice, the animal strain used, and the specific muscle analyzed. In our study, PDX therapy did not significantly influence muscle weight or single fiber CSA; however, a trend towards single fiber hypertrophy in the EDL was observed with treatment.

### ECG

Cardiac aging is associated with left ventricular hypertrophy, diastolic dysfunction, and increased prevalence of atrial fibrillation [91]. As discussed above, aging is also associated with kidney dysfunction [85] and in humans, kidney disorders increase predisposition to cardiac arrythmias, including atrial fibrillation [92]. We saw that, according to the ECG analysis, aging was associated with prolonged QRS time in both sexes. We further reported that old female mice have higher Amp P and lower Amp R, while old male mice had increased T wave duration and lower Amp P and S as compared to Adult. These results are in agreement with previously published works focused on 20-24-month-old mice [93] and when comparing aged mice (24 months) to middle age (12 months) [94], which suggests susceptibility to dysrhythmia [95]. In humans, slow ventricular conduction may result in the prolongation of QRS duration, which is a risk factor for ventricular fibrillation [96, 97]. Yet, translating human ECG interpretations to mice might be challenging, as there are many differences between species regarding heart size, cardiac rate, and age-related comorbidities that are more common in humans and can impact ECG curves [93]. We did not observe any relevant alterations in the electrical signal of the heart with respect to PDX treatment in either male or female animals.

In summary, using a comprehensive approach, our data describe the most relevant age-driven impairments in male and female mice. In our model, aging caused visceromegaly paralleling loss of fat and lean mass and bone mineral density. Aging further led to a dramatic decline in physical performance, facilitating the onset of frailty. In females, there was also evidence of impaired insulin clearance, as well as hepatic activation of pro-inflammatory and Myc pathways and concomitant downregulation of adipogenic and metabolic genes, which was partially reversible with PDX treatment. Importantly, our data supports PDX as a promising anti-frailty therapeutic strategy by its beneficial impact on physical decline and preventive role opposed to bone mineral density loss.

### Supplementary information

ESM 1 Supplemental Figure 1. Experimental design. Experimental groups consisted of Adult vehicle-treated (6 month-old), Old vehicle-treated (22 month-old) and old PDX-treated (22 month-old) female and male C57/BL6 mice at baseline. The experimental timeline was initiated with 2 weeks of acclimation (weeks 1-2), followed by 2 weeks of baseline testing period (weeks 3-4). After baseline, animals were assigned to receive daily gavage of either vehicle or PDX for 9 weeks (gavaging period, weeks 5-13). Endpoint consisted of 2 weeks of testing (weeks 12-13), when the Adult and Old animals were 10 months and 26 months old, respectively. The first week of testing consisted of Walking Speed at day 1, Strength and Endurance at day 2, and Physical Activity from day 3 to day 7. oGTT, ECG and FI tests were performed at week 2 of each Testing period. oGTT: Oral Glucose Tolerance Test; ECG: Electrocardiogram; FI: Frailty Index. (JPG 247 kb)

ESM 2 Supplemental Figure 2. Food intake of female and male mice during experimental timeline. Weekly food intake was monitored throughout the experimental timeline in female (A) and male (B) mice. Briefly, food consumption was calculated by the subtraction of food left from the total food placed in the container. Adult and Old animals were 6 months and 22 months old, respectively, at baseline, and 10 months and 26 months old, respectively, at endpoint. Data are expressed as mean ± SEM. All statistical differences were determined by repeated measures two-way ANOVA with Bonferroni’s *post hoc* test, comparing Old versus Adult and Old versus PDX. *P < 0.05 vs. Adult and ^#^*P* < 0.05 vs. PDX. Arrow indicates the initiation of gavaging period. (JPG 231 kb)

ESM 3 Supplemental Figure 3. Aging is associated with glomerular hypertrophy and mesangial expansion, which was not altered by PDX treatment in females. Kidney was harvested at euthanasia and weighed (A) prior to OCT embedding and freezing. Panel B displays the most representative images of glomerular periodic acid–Schiff (PAS) staining (B) that was used for calculation of glomerular area (C) and mesangial expansion (D) in female mice. Black arrows indicate the glomerulus in each image. EDL and soleus were also harvested at euthanasia and embedded in OCT for cryostat sectioning and histological analysis. Typical images (E) of cross-sectional area (CSA) of H&E stained EDL (F) and soleus (G) indicated muscle fiber size in male and female mice. Data are expressed as mean ± SEM. Differences were determined by Student’s *t* test comparing Old versus Adult and Old versus PDX. **P < 0.01 vs. Adult; ****P < 0.0001 vs. Adult. (JPG 839 kb)

ESM 4 Supplemental Figure 4. Principal Component Analysis (PCA) of hepatic gene expression in female mice. RNA extracted from liver harvested at euthanasia from *n*=5 female mice each from the Adult, Old and PDX-treated groups was sequenced, and counts of uniquely aligned proper read pairs assigned to single Ensembl Gene loci were normalized using the variance stabilizing transformation (VST) from the DESeq2 R package to produce expression values with constant variance and normalized with respect to library size. VST values for each gene were then *z*-normalized across all samples (set to a mean of zero and a standard deviation of one) prior to performing Principal Component Analysis (PCA). All samples were plotted with respect to PC1 (which explains 22% of total variance) and PC2 (which explains 18%). (JPG 194 kb)

ESM 5 Supplemental Figure 5. BMD of male mice. Old- and PDX-treated male mice were euthanized at 25 months of age and tibias were isolated and fixed in 4% formaldehyde. Bone mineral density (BMD) from trabecular (A), cortical (B) and whole bone (C) was analyzed by dual energy X-ray absorptiometry (DEXA). Data are expressed as mean ± SEM. Differences were determined by Student’s *t* test comparing Old versus Adult and Old versus PDX. **P < 0.01 vs. Adult. (JPG 236 kb)

ESM 6 (DOCX 28 kb)

ESM 7 (DOCX 14 kb)

ESM 8 (XLSX 20314 kb)

ESM 9 (DOCX 19 kb)

ESM 10 (XLSX 16 kb)
