# Supplementary figures and images for: Protectin DX as a therapeutic strategy against frailty in mice
Source: GeroScience. 2023 Apr 14;45(4):2601–27. doi: 10.1007/s11357-023-00789-3 (PMC10651819; doi:10.1007/s11357-023-00789-3)

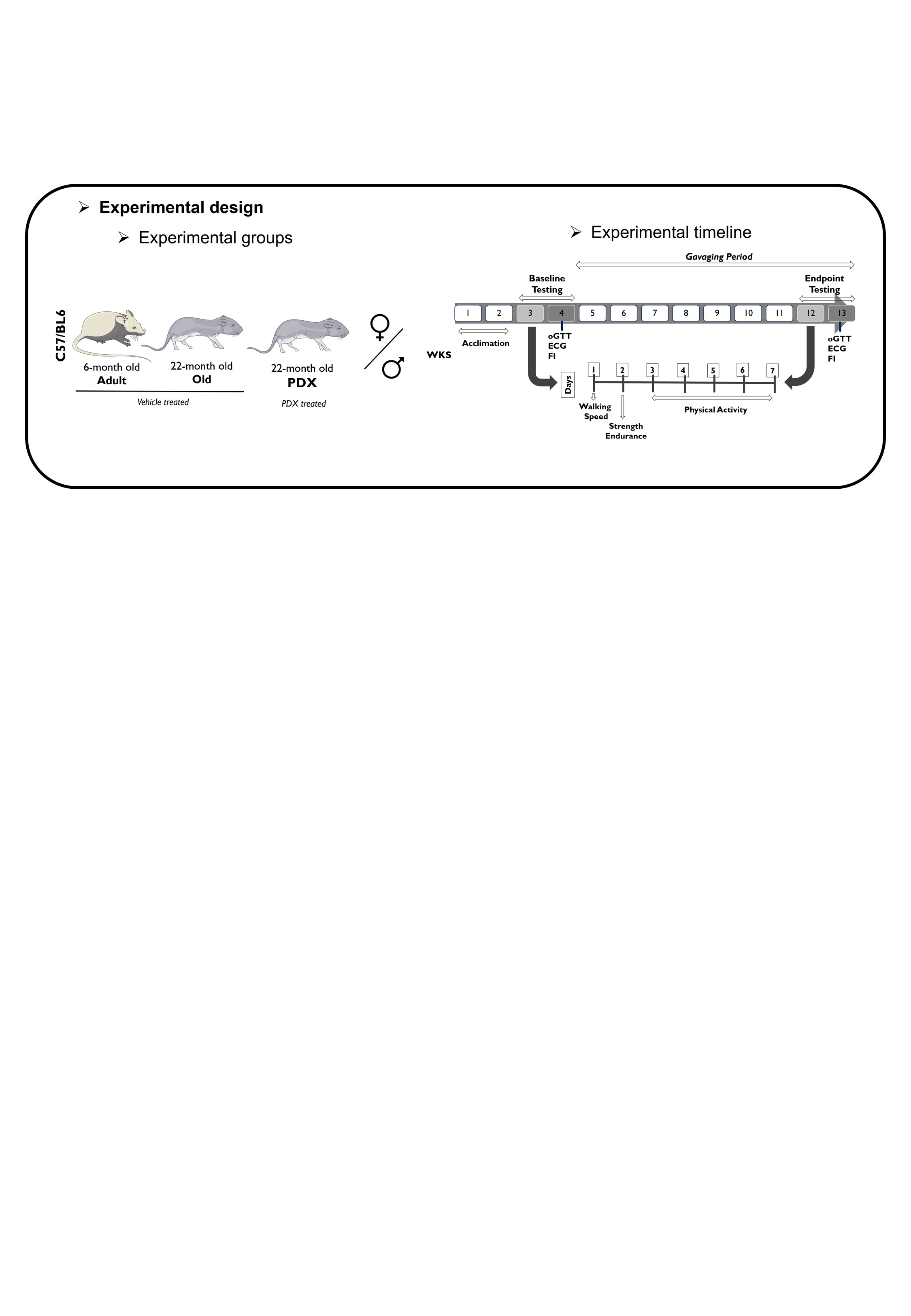

Supplement: Supplementary file 1 — Supplemental Figure 1. Experimental design. Experimental groups consisted of Adult vehicle-treated (6 month-old), Old vehicle-treated (22 month-old) and old PDX-treated (22 month-old) female and male C57/BL6 mice at baseline. The experimental timeline was initiated with 2 weeks of acclimation (weeks 1-2), followed by 2 weeks of baseline testing period (weeks 3-4). After baseline, animals were assigned to receive daily gavage of either vehicle or PDX for 9 weeks (gavaging period, weeks 5-13). Endpoint consisted of 2 weeks of testing (weeks 12-13), when the Adult and Old animals were 10 months and 26 months old, respectively. The first week of testing consisted of Walking Speed at day 1, Strength and Endurance at day 2, and Physical Activity from day 3 to day 7. oGTT, ECG and FI tests were performed at week 2 of each Testing period. oGTT: Oral Glucose Tolerance Test; ECG: Electrocardiogram; FI: Frailty Index. (JPG 247 kb) [file 11357_2023_789_MOESM1_ESM.jpg]

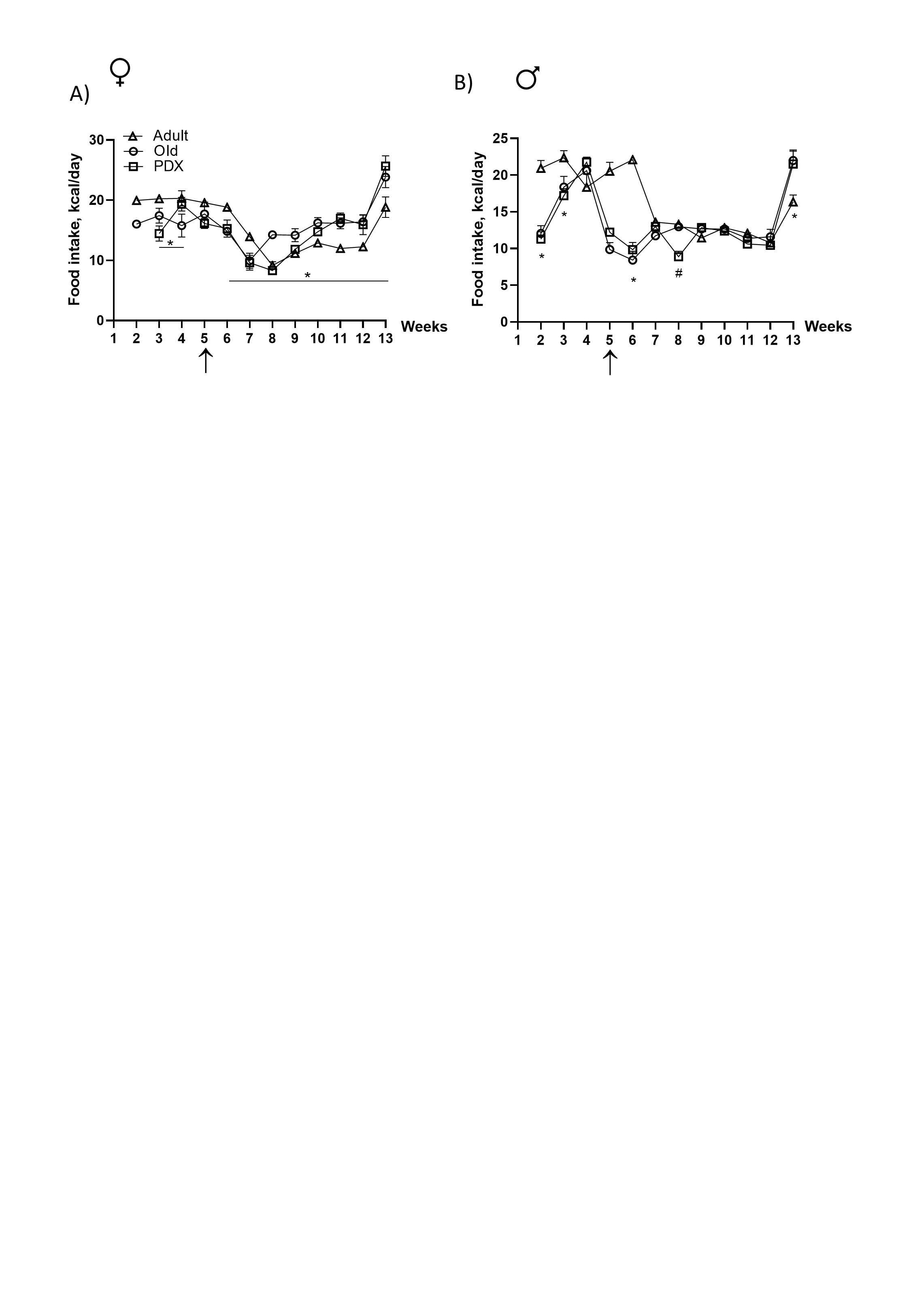

Supplement: Supplementary file 2 — Supplemental Figure 2. Food intake of female and male mice during experimental timeline. Weekly food intake was monitored throughout the experimental timeline in female (A) and male (B) mice. Briefly, food consumption was calculated by the subtraction of food left from the total food placed in the container. Adult and Old animals were 6 months and 22 months old, respectively, at baseline, and 10 months and 26 months old, respectively, at endpoint. Data are expressed as mean ± SEM. All statistical differences were determined by repeated measures two-way ANOVA with Bonferroni’s post hoc test, comparing Old versus Adult and Old versus PDX. *P < 0.05 vs. Adult and #P < 0.05 vs. PDX. Arrow indicates the initiation of gavaging period. (JPG 231 kb) [file 11357_2023_789_MOESM2_ESM.jpg]

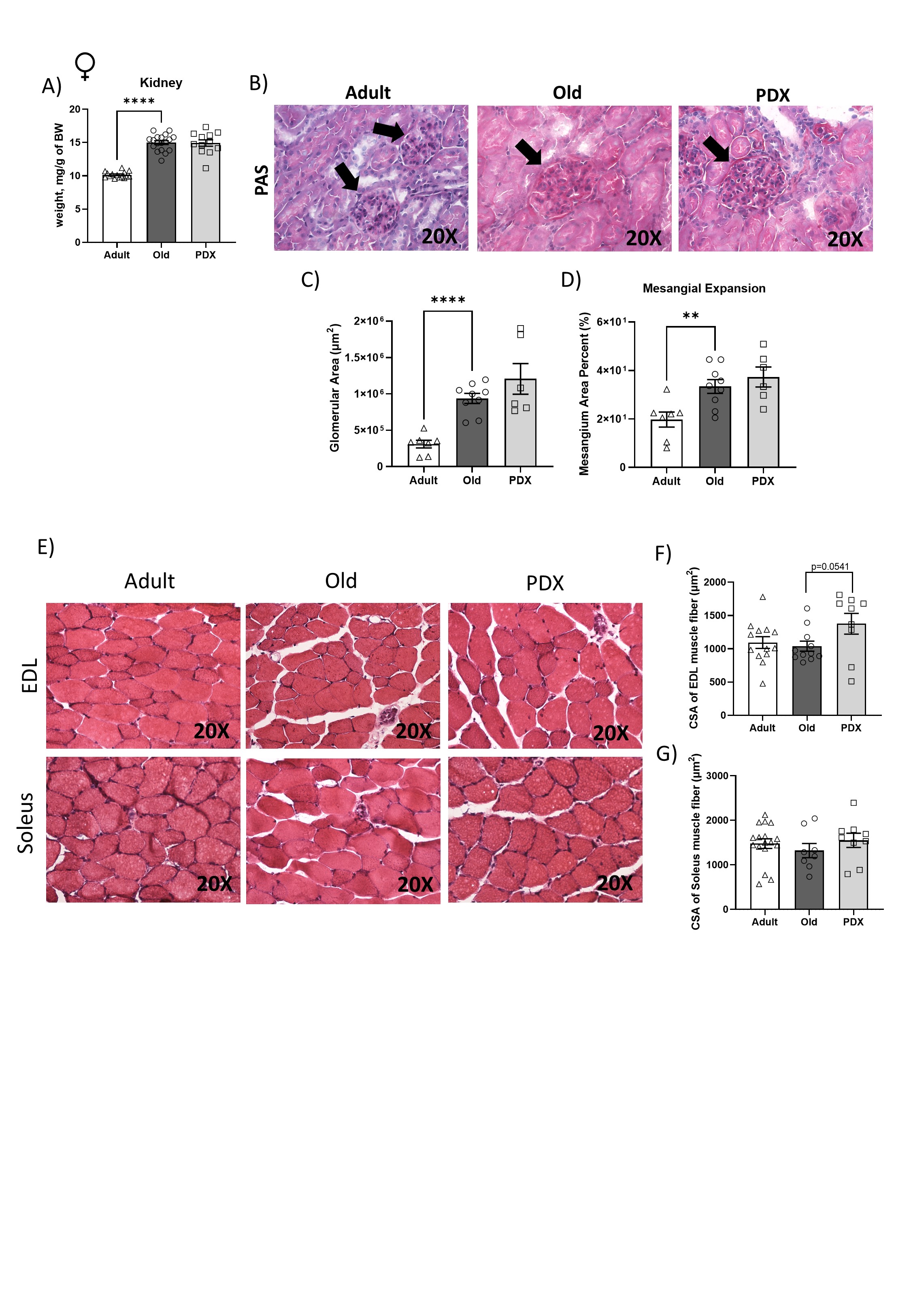

Supplement: Supplementary file 3 — Supplemental Figure 3. Aging is associated with glomerular hypertrophy and mesangial expansion, which was not altered by PDX treatment in females. Kidney was harvested at euthanasia and weighed (A) prior to OCT embedding and freezing. Panel B displays the most representative images of glomerular periodic acid–Schiff (PAS) staining (B) that was used for calculation of glomerular area (C) and mesangial expansion (D) in female mice. Black arrows indicate the glomerulus in each image. EDL and soleus were also harvested at euthanasia and embedded in OCT for cryostat sectioning and histological analysis. Typical images (E) of cross-sectional area (CSA) of H&E stained EDL (F) and soleus (G) indicated muscle fiber size in male and female mice. Data are expressed as mean ± SEM. Differences were determined by Student’s t test comparing Old versus Adult and Old versus PDX. **P < 0.01 vs. Adult; ****P < 0.0001 vs. Adult. (JPG 839 kb) [file 11357_2023_789_MOESM3_ESM.jpg]

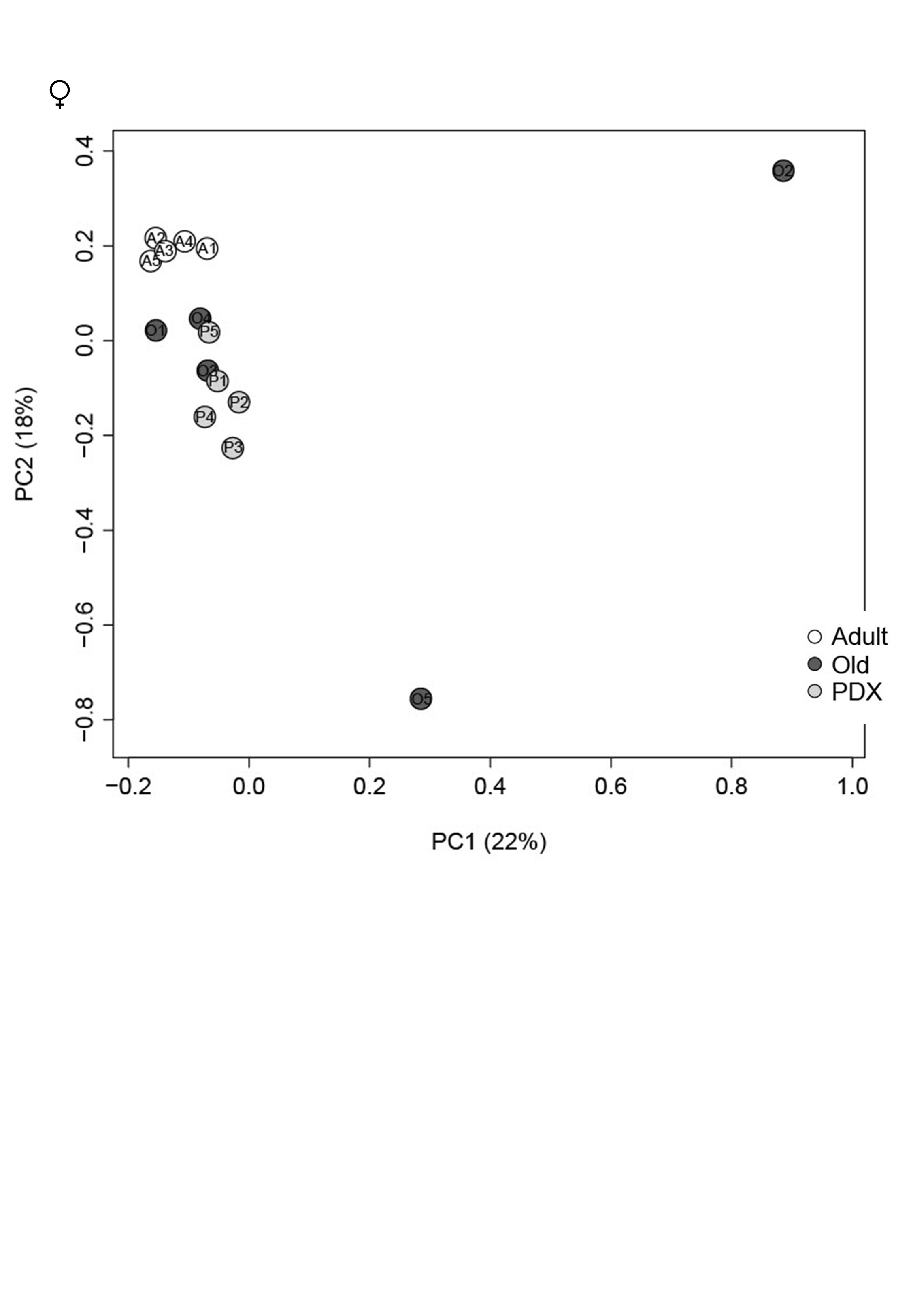

Supplement: Supplementary file 4 — Supplemental Figure 4. Principal Component Analysis (PCA) of hepatic gene expression in female mice. RNA extracted from liver harvested at euthanasia from n=5 female mice each from the Adult, Old and PDX-treated groups was sequenced, and counts of uniquely aligned proper read pairs assigned to single Ensembl Gene loci were normalized using the variance stabilizing transformation (VST) from the DESeq2 R package to produce expression values with constant variance and normalized with respect to library size. VST values for each gene were then z-normalized across all samples (set to a mean of zero and a standard deviation of one) prior to performing Principal Component Analysis (PCA). All samples were plotted with respect to PC1 (which explains 22% of total variance) and PC2 (which explains 18%). (JPG 194 kb) [file 11357_2023_789_MOESM4_ESM.jpg]

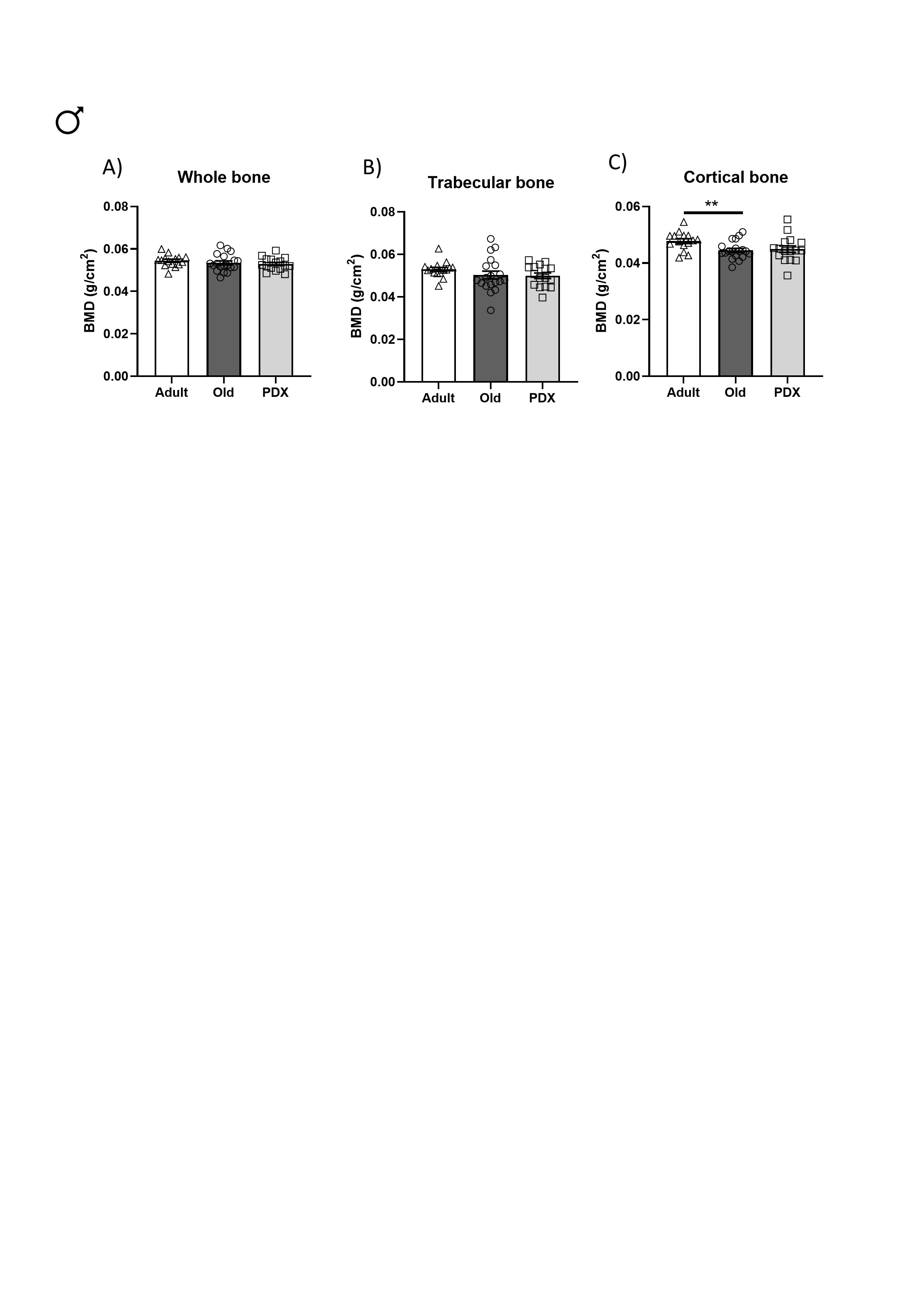

Supplement: Supplementary file 5 — Supplemental Figure 5. BMD of male mice. Old- and PDX-treated male mice were euthanized at 25 months of age and tibias were isolated and fixed in 4% formaldehyde. Bone mineral density (BMD) from trabecular (A), cortical (B) and whole bone (C) was analyzed by dual energy X-ray absorptiometry (DEXA). Data are expressed as mean ± SEM. Differences were determined by Student’s t test comparing Old versus Adult and Old versus PDX. **P < 0.01 vs. Adult. (JPG 236 kb) [file 11357_2023_789_MOESM5_ESM.jpg]
