# Supplementary material for: Protectin DX as a therapeutic strategy against frailty in mice
Source: GeroScience. 2023 Apr 14;45(4):2601–27. doi: 10.1007/s11357-023-00789-3 (PMC10651819; doi:10.1007/s11357-023-00789-3)
Supplement: Supplementary file 6 — (DOCX 28 kb) [file 11357_2023_789_MOESM6_ESM.docx]

|  | **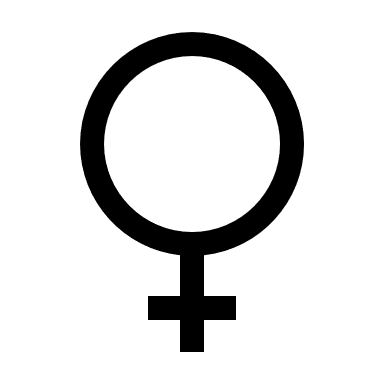** | | | **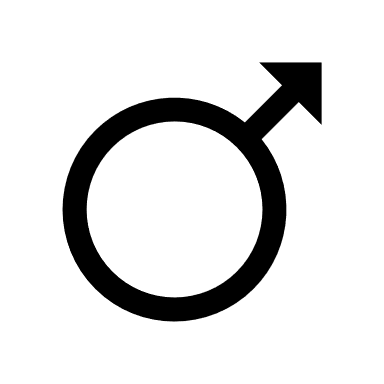** | | |
| --- | --- | --- | --- | --- | --- | --- |
|  | **Adult** | **Old** | **PDX** | **Adult** | **Old** | **PDX** |
| **IFN-γ, pg/ml** | 76.5 ± 50.6 | 52.2 ± 25.5 | 92.6 ± 45.9 | 6.3 ± 1.05 | 35.1 ± 18.3 | 14.0 ± 4.36 |
| **Il-1β, pg/ml** | 17.1 ± 6.03 | 18.0 ± 11.2 | 5.8 ± 0.03 | 6.08 ± 0.31 | 8.54 ± 2.11 | 21.7 ± 6.03 |
| **Il-6, pg/ml** | 6.23 ± 1.65 | 65.4 ± 42.3 | 42.4 ± 15.1 | 9.41 ± 3.45 | **21.8 ± 3.30*** | 28.5 ± 8.23 |
| **Il-10, pg/ml** | 19.7 ± 7.92 | 70.9 ± 26.1 | 23.7 ± 8.78 | 5.79 ± 0.49 | 10.1 ± 3.22 | 24.8 ± 10.9 |
| **MCP-1, pg/ml** | 34.4 ± 6 | **197 ± 49.8*** | 96.1 ± 16.6 | 34.5 ± 10.3 | **105 ± 20.5*** | 73.6 ± 20.5 |
| **RANTES, pg/ml** | 19.5 ± 5.06 | 36.0 ± 12.4 | 18.3 ± 4.99 | 16.5 ± 3.24 | 27.3 ± 5.31 | 26.1 ± 7.04 |
| **TNF-α, pg/ml** | 7.14 ± 0.75 | 29.3 ± 13.2 | 8.10 ± 1 | 6.08 ± 0.04 | **15.5 ± 4.44*** | 8.90 ± 1.66 |

**Supplemental Table 1:** Inflammatory cytokines in plasma of female and male mice. Results are expressed as mean ± SEM. Statistical analysis was assessed by Student's t test, with significant comparisons in boldface: *P < 0.05 vs. Adult within the same sex.
