# Supplementary material for: Protectin DX as a therapeutic strategy against frailty in mice
Source: GeroScience. 2023 Apr 14;45(4):2601–27. doi: 10.1007/s11357-023-00789-3 (PMC10651819; doi:10.1007/s11357-023-00789-3)
Supplement: Supplementary file 7 — (DOCX 14 kb) [file 11357_2023_789_MOESM7_ESM.docx]

| **Sample** | **Total read pairs** | **Aligned reads** | **% of total read pairs with ≥ 1 alignment** | | **Uniquely aligned proper pairs (non-chrM)** | **% of non-chrM uniquely aligned proper pairs** | | |
| --- | --- | --- | --- | --- | --- | --- | --- | --- |
|  |  |  | **chrM** | **rRNA** |  | **Assigned to 1 gene** | **Unassigned** | **Ambiguous (>1 gene)** |
| A1 | 45.704.531 | 96,0% | 3,7% | 0,6% | 38.347.549 | 94,1% | 4,9% | 1,1% |
| A2 | 37.677.838 | 96,3% | 4,2% | 0,6% | 31.451.942 | 94,3% | 4,7% | 1,1% |
| A3 | 28.427.768 | 96,2% | 4,0% | 0,6% | 23.780.946 | 94,3% | 4,6% | 1,1% |
| A4 | 34.679.652 | 96,1% | 4,0% | 0,6% | 29.067.041 | 93,7% | 5,3% | 1,0% |
| A5 | 44.721.787 | 96,4% | 4,0% | 0,6% | 37.529.512 | 94,0% | 4,9% | 1,1% |
| O1 | 34.107.396 | 94,7% | 3,3% | 0,7% | 28.096.088 | 94,8% | 4,1% | 1,0% |
| O2 | 32.569.309 | 95,9% | 2,5% | 0,6% | 27.733.103 | 92,8% | 6,1% | 1,1% |
| O3 | 32.580.209 | 96,0% | 3,7% | 0,6% | 27.293.300 | 93,4% | 5,6% | 1,0% |
| O4 | 36.710.712 | 95,8% | 3,6% | 0,6% | 30.657.690 | 94,8% | 4,2% | 1,0% |
| O5 | 34.209.790 | 96,2% | 4,0% | 0,6% | 28.647.253 | 92,9% | 6,2% | 0,9% |
| P1 | 33.870.713 | 94,1% | 3,2% | 0,7% | 27.663.372 | 94,4% | 4,5% | 1,1% |
| P2 | 34.515.403 | 94,1% | 2,9% | 0,6% | 28.427.034 | 94,5% | 4,5% | 1,0% |
| P3 | 37.213.485 | 95,7% | 3,5% | 0,6% | 31.118.810 | 94,4% | 4,4% | 1,1% |
| P4 | 36.372.286 | 95,7% | 3,3% | 0,6% | 30.467.223 | 94,2% | 4,8% | 1,0% |
| P5 | 37.445.214 | 96,6% | 3,4% | 0,6% | 31.905.287 | 94,5% | 4,5% | 1,0% |

**Supplemental Table 2**: Summary of alignment statistics.
