# Supplementary material for: Protectin DX as a therapeutic strategy against frailty in mice
Source: GeroScience. 2023 Apr 14;45(4):2601–27. doi: 10.1007/s11357-023-00789-3 (PMC10651819; doi:10.1007/s11357-023-00789-3)
Supplement: Supplementary file 9 — (DOCX 19 kb) [file 11357_2023_789_MOESM9_ESM.docx]

|  |  | **Old vs Adult** | | | **PDX vs Old** | | |
| --- | --- | --- | --- | --- | --- | --- | --- |
| **Hallmark gene set name** | **Size** | **NES** | ***p*** | **FDR *q*** | **NES** | ***p*** | **FDR *q*** |
| Allograft Rejection | 185 | 2,50 | < 0.0001 | **0,0000** | -1,12 | 0,2226 | 0,4595 |
| IL-6/Jak/Stat3 Signaling | 81 | 2,43 | < 0.0001 | **0,0000** | -1,34 | 0,0565 | 0,1330 |
| Inflammatory Response | 183 | 2,18 | < 0.0001 | **0,0000** | -1,21 | 0,0963 | 0,2594 |
| Epithelial-Mesenchymal Transition | 187 | 2,14 | < 0.0001 | **0,0000** | -1,62 | < 0.0001 | **0,0060** |
| TNFa Signaling Via NFkB | 192 | 2,13 | < 0.0001 | **0,0000** | -1,12 | 0,2263 | 0,4517 |
| E2F Targets | 195 | 2,03 | < 0.0001 | **0,0000** | -2,23 | < 0.0001 | **0,0000** |
| G2M Checkpoint | 188 | 1,97 | < 0.0001 | **0,0000** | -2,33 | < 0.0001 | **0,0000** |
| KRAS Signaling, Up | 195 | 1,97 | < 0.0001 | **0,0000** | -0,98 | 0,5447 | 0,7353 |
| Interferon Gamma Response | 179 | 1,87 | < 0.0001 | **0,0001** | 1,95 | < 0.0001 | **0,0004** |
| Angiogenesis | 36 | 1,86 | 0,0003 | **0,0001** | -1,33 | 0,1027 | 0,1342 |
| Complement | 178 | 1,81 | < 0.0001 | **0,0004** | 1,11 | 0,1186 | 0,3293 |
| Myc Targets V2 | 57 | 1,77 | 0,0005 | **0,0007** | -2,33 | < 0.0001 | **0,0000** |
| IL-2/Stat5 Signaling | 193 | 1,76 | < 0.0001 | **0,0006** | -0,89 | 0,7520 | 0,8824 |
| Apoptosis | 158 | 1,71 | < 0.0001 | **0,0014** | -1,02 | 0,4486 | 0,6597 |
| Coagulation | 129 | 1,70 | 0,0001 | **0,0013** | -0,80 | 0,8627 | 0,9340 |
| Unfolded Protein Response | 110 | 1,67 | 0,0002 | **0,0019** | -1,45 | 0,0077 | **0,0448** |
| Hypoxia | 192 | 1,60 | 0,0002 | **0,0048** | -1,29 | 0,0374 | 0,1670 |
| Mitotic Spindle | 194 | 1,48 | 0,0021 | **0,0209** | -1,70 | < 0.0001 | **0,0023** |
| Apical Junction | 185 | 1,45 | 0,0047 | **0,0276** | -1,07 | 0,3471 | 0,6038 |
| TGF Beta Signaling | 52 | 1,44 | 0,0357 | **0,0295** | -1,28 | 0,1143 | 0,1634 |
| Myc Targets V1 | 190 | 1,41 | 0,0076 | **0,0380** | -1,89 | < 0.0001 | **0,0001** |
| p53 Pathway | 193 | 1,36 | 0,0167 | 0,0606 | -1,25 | 0,0506 | 0,1954 |
| Estrogen Response Late | 188 | 1,34 | 0,0220 | 0,0698 | -0,68 | 0,9787 | 0,9885 |
| PI3K/Akt/mTOR Signaling | 103 | 1,31 | 0,0686 | 0,0923 | -1,03 | 0,4416 | 0,6707 |
| Myogenesis | 193 | 1,31 | 0,0347 | 0,0912 | -1,27 | 0,0424 | 0,1825 |
| Estrogen Response Early | 186 | 1,29 | 0,0485 | 0,1083 | -0,59 | 0,9970 | 0,9908 |
| mTORC1 Signaling | 197 | 1,27 | 0,0526 | 0,1210 | -0,93 | 0,6729 | 0,8298 |
| UV Response, Up | 147 | 1,26 | 0,0766 | 0,1228 | -1,01 | 0,4706 | 0,6715 |
| Interferon Alpha Response | 84 | 1,20 | 0,1750 | 0,2064 | 3,07 | < 0.0001 | **0,0000** |
| Hedgehog Signaling | 35 | 1,16 | 0,2487 | 0,2603 | -1,06 | 0,3951 | 0,5995 |
| Androgen Response | 96 | 1,07 | 0,3548 | 0,4548 | -0,98 | 0,5337 | 0,7184 |
| KRAS Signaling, Down | 173 | 1,05 | 0,3686 | 0,4837 | -0,82 | 0,8501 | 0,9576 |
| UV Response, Down | 143 | 1,04 | 0,3962 | 0,4938 | -1,56 | 0,0005 | **0,0124** |
| Spermatogenesis | 116 | 1,04 | 0,4048 | 0,4927 | -1,31 | 0,0525 | 0,1465 |
| Cholesterol Homeostasis | 71 | 1,01 | 0,4555 | 0,5319 | 2,34 | < 0.0001 | **0,0000** |
| Glycolysis | 195 | 0,99 | 0,5125 | 0,5744 | -0,80 | 0,8964 | 0,9567 |
| Apical Surface | 41 | 0,95 | 0,5556 | 0,6580 | -0,76 | 0,8371 | 0,9581 |
| DNA Repair | 145 | 0,82 | 0,8250 | 0,8834 | -1,22 | 0,1007 | 0,2421 |
| Heme Metabolism | 186 | 0,78 | 0,9019 | 0,9190 | -1,34 | 0,0133 | 0,1399 |
| Wnt/Beta-catenin Signaling | 39 | 0,70 | 0,8974 | 0,9786 | -1,16 | 0,2653 | 0,3808 |
| Protein Secretion | 91 | 0,59 | 0,9952 | 0,9945 | -0,83 | 0,7963 | 0,9826 |
| Pancreas Beta Cells | 33 | -1,05 | 0,3560 | 0,3221 | 0,89 | 0,6732 | 0,7241 |
| Notch Signaling | 30 | -1,19 | 0,1981 | 0,1361 | 0,93 | 0,5818 | 0,6810 |
| Reactive Oxygen Species Pathway | 48 | -1,34 | 0,0624 | 0,0508 | 0,95 | 0,5628 | 0,6971 |
| Xenobiotic Metabolism | 196 | -2,23 | < 0.0001 | **0,0000** | 1,61 | < 0.0001 | **0,0113** |
| Peroxisome | 103 | -2,31 | < 0.0001 | **0,0000** | 1,08 | 0,2426 | 0,3724 |
| Adipogenesis | 195 | -2,66 | < 0.0001 | **0,0000** | 1,77 | < 0.0001 | **0,0028** |
| Fatty Acid Metabolism | 152 | -2,82 | < 0.0001 | **0,0000** | 1,58 | < 0.0001 | **0,0122** |
| Bile Acid Metabolism | 110 | -2,93 | < 0.0001 | **0,0000** | 2,27 | < 0.0001 | **0,0000** |
| Oxidative Phosphorylation | 189 | -3,32 | < 0.0001 | **0,0000** | 2,08 | < 0.0001 | **0,0000** |

**Supplemental Table 4.** Gene Set Enrichment Analysis (GSEA) performed using Hallmark gene sets in Old vs Adult and PDX vs Old comparisons. Gene sets with positive or negative NES values are coordinately upregulated or downregulated, respectively, with respect to a given comparison. Results with FDR q < 0.05 are highlighted in boldface.
